# Dynamics of zoosporic parasites in summer phytoplankton communities of the Baltic Sea

**DOI:** 10.1093/femsec/fiaf081

**Published:** 2025-08-07

**Authors:** Silke Van den Wyngaert, Ali Nawaz, Elisabet Alacid, Steffaney M Wood-Rocca, Albert Reñé, Esther Garcés, Anke Kremp, Christian Wurzbacher

**Affiliations:** Department of Biology, University of Turku, Vesilinnantie 5, 20014 Turku, Finland; Bioinformatics, Department of Chemistry and Biology, University of Siegen, Am Eichenhang 50, 57076 Siegen, Germany; Department of Marine Ecology, Centre for Advanced Studies of Blanes (CEAB), CSIC, 17300 Blanes, Catalonia, Spain; Center for Marine Biotechnology and Biomedicine, Scripps Institute of Oceanography, University of California San Diego, La Jolla, CA 92093, United States; Microbial and Environmental Genomics, J. Craig Venter Institute, La Jolla, CA 92037, United States; Institut de Ciències del Mar (ICM-CSIC), Dpt. Biologia Marina i Oceanografia, Passeig Marítim de la Barceloneta, 37-49, 08003 Barcelona, Catalonia, Spain; Institut de Ciències del Mar (ICM-CSIC), Dpt. Biologia Marina i Oceanografia, Passeig Marítim de la Barceloneta, 37-49, 08003 Barcelona, Catalonia, Spain; Biology Department, Leibniz Institute for Baltic Sea Research Warnemuende, Seestr. 15, 18119 Rostock, Germany; Chair of Urban Water Systems Engineering, Technical University of Munich, Am Coulombwall 3, 85748 Garching, Germany

**Keywords:** Baltic Sea, chytrids, Illumina, metabarcoding, microbial eukaryotes, Oxford Nanopore Technologies, parasitism, phytoplankton

## Abstract

Zoosporic parasites significantly influence aquatic ecosystems by infecting various phytoplankton taxa, but their interactions in brackish ecosystems remain largely unexplored. This study explores microbial communities and parasitic interactions with summer phytoplankton communities at six brackish coastal sites in the northern Baltic Sea. We hypothesized that small-scale spatial heterogeneity in environmental conditions would lead to distinct assemblages of microbial communities and phytoplankton–parasite interactions. By combining DNA metabarcoding, single-cell sequencing, and microscopy, we provide the first community-level qualitative and quantitative assessment of zoosporic parasites infecting summer phytoplankton in the Baltic Sea. Microbial communities varied significantly across sites, with salinity as primary driver of eukaryotic diversity. Chytrid fungi were the dominant parasites, infecting green algae, diatoms, and filamentous cyanobacteria, with infection rates up to 5.8% of phytoplankton biomass. Sequences from brackish chytrids clustered with those from freshwater environments, reflecting polyphyletic patterns linked to host taxa. Phytoplankton–parasite interactions were influenced by host abundance and site-specific conditions with correlation analysis suggesting broader host ranges and potential generalist behavior in some chytrid species. Additionally, an unidentified oomycete infected up to 85% of the toxic bloom-forming cyanobacterium *Nodularia* spp. This study highlights the ecological relevance of zoosporic parasites in the Baltic Sea and emphasizes the need for further research into their role in phytoplankton bloom dynamics.

## Teaser

This first community-level assessment of phytoplankton–zoosporic parasite interactions in the Baltic Sea highlights their diversity, abundance, and potential role in regulating harmful algal blooms and shaping phytoplankton communities.

## Introduction

Microbial interactions are fundamental to ecosystem processes, yet the extremely high diversity of aquatic microbes means many of these interactions likely remain uncharacterized, limiting our understanding of their ecological roles. Parasitism stands out as the most common consumer strategy among organisms (Lafferty et al. 2008). In aquatic ecosystems, parasitic interactions between protists are abundant, and likely underestimated compared to predator–prey interactions (Bjorbækmo et al. 2020). Zoosporic parasites, which infect a broad range of phytoplankton taxa (Jephcott et al. 2016, Frenken et al. 2017), share common life cycle features, including a host-associated vegetative stage and free-living motile infective stages (e.g. zoospores). By causing lethal infections, these parasites are also referred to as parasitoids, and they can exert direct top-down control on phytoplankton populations (Ibelings et al. 2011), including harmful algal blooms (Alacid et al. 2017, Gerphagnon et al. 2017) or benefit host populations by selectively parasitizing weaker, unhealthy individuals (Laundon et al. 2021). Beyond population-level effects, zoosporic parasites mediate aquatic food webs by enhancing herbivory (Frenken et al. 2020, Rasconi et al. 2020) and accelerating carbon transfer to higher trophic levels (Kagami et al. 2014, Klawonn et al. 2021). Through the fungal shunt, chytrid infections divert phytoplankton-derived carbon into zoospores, which are readily consumed by grazers, thereby bypassing the microbial loop (Klawonn et al. 2021). Moreover, chytrid infections have the potential to diminish the strength and efficiency of vertical organic matter fluxes by reducing the formation of diatom aggregates, enhancing remineralization, and consequently decreasing carbon export via sedimentation (Klawonn et al. 2023a).

Research on zoosporic parasites has traditionally focused on different taxa depending on the environment, likely reflecting the distinct distribution and abundance of specific parasite groups in freshwater and marine ecosystems. Chytrids have been studied more extensively in freshwater (Canter 1954, Frenken et al. 2017, Gsell et al. 2022), with the majority of currently described parasitic chytrid species originating from temperate lakes (Van den Wyngaert et al. 2017, 2018, Seto and Degawa 2018, Seto et al. 2020). In contrast, nonfungal, eukaryotic phytoplankton parasites, such as Perkinsozoans, Pirsoniales, Cercozoa, and Labyrinthulomycetes, are better studied in marine environments (Kuhn and Hofmann 1999, Norén et al. 1999, Hassett 2020, Reñé et al. 2021). The application of high-throughput sequencing has revealed greater complexity in microbial diversity. While Chytridiomycota usually dominate fungal communities in pelagic freshwater ecosystems (Panzer et al. 2015, Van den Wyngaert et al. 2022), they can also be abundant in marine environments (Richards et al. 2015, Comeau et al. 2016), particularly in environments with atypical salinity such as sea ice, the Baltic Sea, or the Red Sea (Hassett et al. 2020). Moreover, marine sediments often harbor increased diversity of chytrids (Fernández-Valero et al. 2022, Reñé et al. 2023), possibly serving as a habitat for both active benthic species and a reservoir for resting spores, which are observed in the life cycle of several obligate parasitic chytrids (van Donk and Ringelberg 1983, Van den Wyngaert et al. 2017, Seto et al. 2020). Additionally, observations of phytoplankton–chytrid interactions are increasing across marine ecosystems (Hassett and Gradinger 2016, Garvetto et al. 2019, Kilias et al. 2020, Karpov et al. 2021, Fernández-Valero et al. 2024, Ilicic et al. 2024). Notably, a recent study demonstrated that chytrids and Perkinsozoa can coinfect the same dinoflagellate host population in the Baltic Sea, occupying similar niches (Reñé et al. 2022). Despite these advances, few studies combine next-generation sequencing with empirical observations of phytoplankton–parasite interactions (Fernández-Valero et al. 2022, Reñé et al. 2023), and even fewer integrate quantitative data on these interactions (but see Van den Wyngaert et al. 2022, Fernández-Valero et al. 2023). Moreover, zoosporic parasites remain poorly characterized, with a limited number of reference sequences hindering our ability to link zoosporic sequence diversity to consumer–resource interactions.

The Baltic Sea is one of the largest brackish water bodies in the world. It has a unique and dynamic environment characterized by a pronounced horizontal salinity gradient, ranging from almost freshwater in the northern Bothnian Bay to almost full marine conditions in the Danish Straits. In recent decades, the northern Baltic Sea has experienced significant changes in phytoplankton communities, with a marked increase in late summer biomass, largely driven by changing climate-conditions and eutrophication. This increase is particularly evident in the proliferation of cyanobacteria, as well as various flagellate taxa, such as dinoflagellates and prymnesiophytes (Suikkanen et al. 2007, 2013, Karlson et al. 2021). Cyanobacteria-dominated communities, which are known to produce toxins and are a low quality food source for many grazers, have profound implications for the entire ecosystem. In this context, studying parasitic interactions during these summer blooms is highly relevant, as they may influence the dynamics of these increasingly common and ecologically significant blooms.

The primary objectives of our study were (i) to investigate the diversity of microbial communities and zoosporic parasite interactions with summer phytoplankton communities in heterogeneous, brackish coastal sites in the northern Baltic Sea, and (ii) to provide the first quantitative data on phytoplankton infections in the Baltic Sea. To achieve this, we employed DNA metabarcoding, long-read sequencing of manually isolated infected single phytoplankton cells or colonies, and microscopic observation. The sampling sites were chosen to capture the environmental heterogeneity of the Northern Baltic coasts, particularly in salinity, enabling us to investigate how abiotic factors shape microbial communities and host–parasite interactions. This spatial contrast also increased the likelihood of capturing a broader range of phytoplankton and fungal (including parasitic) diversity across different ecological contexts. We hypothesized that the small-scale spatial heterogeneity in environmental conditions would lead to distinct and diverse assemblages of microbial communities and phytoplankton–parasite interactions.

## Material and methods

### Study site and sample collection

We sampled six coastal sites in the Tvärminne area, southwest coast of Finland on three occasions during summer (20 July, 27 July, and 7 August 2018). Three sites were located in the shallow, semienclosed and sheltered innermost coastal zone (Stadsfjärden and the end of Pojoviken fjord), near the village Ekenäs (referred to as INNER sites). The other three sites were in the more exposed outer archipelago zone, around 25 km southwest at the mouth of the Gulf of Finland, near Tvärminne Zoological Station (referred to as OUTER sites) (Fig. S1). The zones were defined according to Niemi (1973). Surface water samples were prefiltered through a 200 µm nylon mesh to remove larger mesozooplankton. A subsample was used for isolation of infected single phytoplankton cells/colonies for Nanopore sequencing as described in Wurzbacher et al. (2019). Volumes of 200–700 ml of water samples were size-fractionated and collected onto 5 and 0.2 µm pore size polycarbonate filters (47 mm diameter, Merck Millipore, Germany) for DNA metabarcoding of the fungal and eukaryotic communities. All filters were stored in cryotubes at −80°C until further processing. Sediment samples were taken with a limnos sediment corer and the topmost 1 cm of the sediment was collected and stored at −80°C until further processing for DNA metabarcoding. Lugol-fixed samples were preserved for the quantification of phytoplankton (acidic Lugol’s solution) and fungal parasites (neutral Lugol’s solution). Additional samples were taken for Chlorophyll a (Chl *a*) and nutrient analysis in acidified plastic bottles.

### Measurement of physico-chemical factors

Environmental parameters (temperature, oxygen, salinity, and pH) were measured *in situ* with a multiprobe (YSI ProSolo ODO/CT, Xylem). For chlorophyll a (Chl *a*), 50 ml of water was filtered on GF/F filters and Chl *a* was extracted in 96% ethanol. Chl *a* concentration was measured with a spectrofluorometer (Varian Cary Eclipse fluorescence spectrophotometer, excitation 430 nm, emission 670 nm). Nutrients were measured by colorimetric methods (Grasshoff et al. 1999) with a photometric analyser (Thermo Scientific Aquakem 250). Phosphate (PO_4_) was analyzed using the antimony–molybdate method, silicon (Si) using the molybdate-blue method and nitrite (NO_2_) by the formation of azo-dye. For the analysis of total phosphorus and total nitrogen, peroxodisulphate was used for the oxidation of organic material. Ammonium (NH_4_) was analyzed using the indophenol blue method and measured with a Shimadzu Hitachi 1100 spectrophotometer.

### Microscopy of phytoplankton and parasite infections

Phytoplankton samples were analyzed using the Utermöhl method (Utermöhl 1958) under an inverted microscope (M3 Leitz DMIRB), according to the HELCOM monitoring guidelines (HELCOM 2021). A volume of 5, 10, 25, or 50 ml (depending on the abundance of cells) was settled in an Utermöhl settling chamber. For further details regarding microscopy, see e.g. Lehtinen et al. (2016). The observed taxa were determined to the most detailed possible taxonomic level, and the specimens were further categorized into size classes. Cell counts were converted into biomass (µg l^−1^), using the taxon-specific counting units, size classes, and biovolume formulae used by the HELCOM EG PHYTO (Expert Group on phytoplankton) (Olenina et al. 2006) and its annually updated annex.

For the quantification of parasite infections, subsamples of 50 ml were concentrated by overnight cell sedimentation in 50 ml centrifugation tubes and subsequent removal of the upper 45 ml. Two ml of the concentrated cell suspension were dual-stained with the chitin-binding dyes Calcofluor White (CFW) and Wheat Germ Agglutinin conjugated to Alexa Fluor 488 (WGA-488), according to Klawonn et al. (2023b). This staining protocol specifically targeted the detection of fungal parasites. The sample was prescreened for infected phytoplankton taxa using an inverted microscope (Nikon eclipse Ti2, 400X, fluorescence channels CFW: 387/11 nm excitation and 442/46 nm emission, WGA: 482/35 nm excitation and 536/40 nm emission). If infections were detected on a phytoplankton species, 200 cells (in the case of single-celled species) or colonies of the target species were counted to determine the proportion of infected cells (e.g. the prevalence of infection). For some samples, this threshold was not reached due to low biomass. Infected phytoplankton host biomass was calculated by multiplying the host biomass by the proportion of infected host cells (Gsell et al. 2022).

### Isolation, whole genome amplification, fungal ribosomal operon library, and long-read sequencing of infected single cells/colonies

Individual phytoplankton cells/colonies with visible parasite infections were manually picked and washed in 0.2 µm filtered artificial seawater (f/2 medium adjusted to the salinity of the sites) using a micropipette under an inverted light microscope (Leica DMIRB) as described in Van den Wyngaert et al. (2022). DNA of single infected cells was extracted and amplified using Illustra Single Cell GenomiPhi DNA amplification kit (GE-Healthcare).

To taxonomically identify the fungal parasites isolated from infected phytoplankton cells, the complete fungal ribosomal operon (FRO) (SSU-ITS1-5.8S-ITS2-LSU rRNA) was amplified using the primer pair NS1short (5′-CAGTAGTCATATGCTTGTC-3′) and RCA95 m (5′-CTATGTTTTAATTAGACAGTCAG-3′) (Wurzbacher et al. 2019). The polymerase chain reaction (PCR) amplifications were carried out in a 25 µl reaction volume containing PrimeSTAR GXL polymerase master mix (TaKaRa Bio, Shiga, Japan), 10 pmol of each primer and 2 µl of gDNA (i.e. MDA product) using the following reaction conditions: initial denaturation at 98°C for 10 min, 36 cycles at 98°C for 10 s, 55°C for 15 s, and a final extension step of 150 s at 68°C.

Prior to the amplicon library preparation, the PCR products were visualized and the fragment lengths (ribosomal operon: ∼4–6 kb) were validated through electrophoresis on a 1% (w/v) agarose gel. Positive PCR products were then purified using 0.8 v/v of AMPure-XP beads (Beckmann), following the manufacturer’s guidelines. Subsequently, the purified PCR products were barcoded with unique molecular identifiers, which involved specific index primer combinations for each amplified fungal region. The indexed PCR products underwent a second purification step, using 0.8 v/v of AMPure beads (Beckmann). Following this, the purified indexed PCR products were quantified using the DeNovix broad range dsDNA Assay (DeNovix Inc., Wilmington, Germany), following the manufacturer’s instructions. After quantification, the PCR products were pooled in equimolar quantities, and the concentration of the resulting pool was determined using the DeNovix broad-range dsDNA Assay (DeNovix Inc.). Finally, 1 µg of the pooled DNA was used for the preparation of an Oxford Nanopore library, following the manufacturer’s protocol and the recommendations for the SQK-LSK112 library preparation kits (Oxford Nanopore Technologies—ONT).

For single cell samples where long-read sequencing failed, fragments of the LSU and SSU rRNA genes of chytrids were amplified as previously described by Van den Wyngaert et al. (2022), and Sanger sequenced by Macrogen Europe.

### Phylogenetic analysis

To clarify the phylogenetic position of the single cell isolates, a phylogenetic analysis was conducted using a data set comprising partial LSU rRNA and Nanopore generated long-read reference sequences, along with the sequences of the single-cell isolates obtained in this study. Sequences were aligned using MAFFT v7.487 (Katoh and Standley 2013). The LSU rRNA sequences were first aligned separately and the long-read sequences were added into the reference alignment using MAFFT with the “–addlong” option. The alignment was trimmed using trimAl (Capella-Gutiérrez et al. 2009) with the “gappyout” method. The maximum likelihood tree was inferred with IQ-TREE 2 (Minh et al. 2020). The best model of the alignment was examined using ModelFinder (Kalyaanamoorthy et al. 2017) implemented in IQ-TREE 2. According to the corrected Akaike information criterion, GTR + F + R5 was the best-fit model. The tree was visualized with FigTree (https://github.com/rambaut/figtree) and edited with Inkscape (https://www.inkscape.org).

### DNA isolation, metabarcoding, and Illumina sequencing of fungal and microbial eukaryotic communities

For metabarcoding of water and sediment samples, genomic DNA was extracted using a CTAB–phenol–chloroform–isoamylalcohol/bead-beating protocol modified after Nercessian et al. (2005). Briefly, for water samples, filters were homogenized in a bead beater using glass/zirconia beads (0.1, 0.7, and 3 mm), plus 600 µl extraction buffer (10% CTAB in 1.6 M NaCl mixed 1:1 with 240 mM K_2_HPO_4_/KH_2_PO_4_ buffer), 600 µl phenol:chloroform:isoamylalcohol (25:24:1) and 60 µl each 10% sodium dodecyl sulfate and 10% *N*-lauroyl sarcosine. The same extraction protocol was used for sediment samples, with 0.6 g of sediment as starting material. The aqueous phase was mixed with an equal volume of chloroform–isoamylalcohol (24:1) to remove any residual phenol. DNA was precipitated with 30% polyethylene glycol (PEG) 6000 in 1.6 M NaCl and the addition of 1 µl linear polyacrylamide (LPA) (Sigma), followed by a centrifugation step. The resulting pellet was washed with 1 ml ice-cold ethanol (70%), dried briefly at 37°C, resuspended in 50 µl diethyl pyrocarbonate (DEPC)-treated water, and stored at −80°C until further processing. DNA extraction was successful for only nine sediment samples.

PCR, library preparation, and sequencing were performed by LGC Genomics (Berlin, Germany). For targeting fungi, the D1 region of the Large Subunit (LSU) rRNA was amplified using the forward primer ITS4ngsF (5′-GCATATCAATAAGCGSAGGA3′) and the reverse primer LF402R (5′-TTCCCTTTYARCAATTTCAC-3′) (modified after Tedersoo et al. 2015). For eukaryotes, the V4 region of the SSU rRNA was amplified using the forward primer TAReuk454FWD1 (5′-CCAGCA(G/C)C(C/T)GCGGTAATTCC-3′) and the reverse primer TAReukREV3 (5′-ACTTTCGTTCTTGAT(C/T) (A/G)A-3′) (Stoeck et al. 2010). PCR was followed by library preparation and sequencing (2 × 300 bp) on a MiSeq (Illumina) platform.

### Bioinformatic analysis of Illumina and nanopore sequences

The bioinformatics data processing of amplicon raw sequences from fungal and eukaryotic communities (based on metabarcoding) was carried out using the dadasnake pipeline (a snakemake implementation of DADA2) (Weißbecker et al. 2020). Briefly, sequences corresponding to the forward and reverse primers were trimmed from the demultiplexed raw reads using “cutadapt” (Martin 2011). Reads were filtered, trimmed, and merged with the merging criteria of a minimum overlap of 12 nucleotides and no mismatch. After chimera removal, we obtained 1 720 231 fungal reads and 1 272 994 eukaryotic reads, clustered into 3067 and 3070 amplicon sequence variants (ASVs), respectively. Three out of the nine sediment samples had to be discarded due to a low read output. Subsequently, the representative reads from each ASV were annotated against SILVA database v138 (Quast et al. 2013) for taxonomic classification. In addition, we compared ASVs from the fungal dataset to a custom reference library of parasitic chytrid species of phytoplankton, including the generated sequences of single-cell isolates from this study. An ASV was considered identical to a sequence when reaching a sequence similarity of ≥99% and a minimum sequence coverage of 85%.

For processing the Oxford Nanopore long-read sequences of the complete FRO, we employed a bioinformatic workflow outlined in Wurzbacher et al. (2019). This process involved initial quality filtering with a maximum error rate of 0.03 using USEARCH v8.1 (Edgar 2010), length-based filtering with Biopython v1.65 (Cock et al. 2009), and demultiplexing with Flexbar v2.5 (Dodt et al. 2012) based on unique barcodes. Subsequent steps included sequence alignment using MAFFT v7.397 (Katoh and Standley 2013), clustering with the Opticlust algorithm in mothur v1.39 (Schloss et al. 2009), and generating consensus sequences with Consension (v1.0, https://microbiology.se/software/consension/). A clustering threshold of 0.03 was applied, and, in cases of multiple OTUs, the most abundant OTU was chosen for downstream sequence similarity comparisons.

Raw sequence data of fungal and eukaryotic metabarcoding data were deposited into the NCBI Sequence Read Archive under the Bioproject number PRJNA1130152. Sequences from single cell/colony isolates were deposited under the Bioproject number PRJNA1218728 (Nanopore long reads) and under accession number PV053655 (SSU rRNA Sanger), PV053652–PV053654 (LSU rRNA Sanger), and are listed in Supplementary file S1.

### Statistical analysis

Prior to multivariate statistical analysis on fungal community data, all the ASVs annotated as nonfungi and with low prevalence (present in <4% of samples) were filtered out, resulting in a dataset of 864 fungal ASVs for downstream analysis. To statistically test the impact of removing low-prevalence ASVs, we computed Bray–Curtis distance matrices for the full and filtered datasets and performed a Mantel test (Spearman’s ρ = 0.95, *P* < .001). The high correlation indicates that the removal of low-prevalence ASVs from the dataset did not affect the community composition for downstream analysis. Similarly, in the dataset representing microbial eukaryotes, the removal of nontargeted and low-prevalence taxa resulted in 1135 ASVs, to be used in subsequent analysis.

All statistical analyses were carried out within the *R environment* v3.0 (R Core Team 2022) and PAST software (Hammer et al. 2001). The environmental data matrix (abiotic factors) was standardized using the “*decostand*” function of the vegan package (Oksanen et al. 2022). The sampling effort curves from all the samples in the respective datasets were generated using the “*rarefy*” function of the vegan package (Fig. S2). The alpha diversity of eukaryotic and fungal taxa across INNER and OUTER sites and size fractions was calculated using the Shannon’s diversity index. The differences in the alpha diversity among the groups were tested using Kruskal–Wallis test, followed by the *post hoc* pairwise comparisons using Dunn’s test from “microeco” R package (Liu et al. 2021).

To visualize the patterns in microbial communities from INNER and OUTER site samples, we applied principal coordinate analysis (PCoA) using Bray–Curtis distance measure using the vegan package in R. To assess the significance of differences in microbial communities between INNER and OUTER sites, and to evaluate the effect of different variables (salinity, sampling date, and filter size), we conducted permutational multivariate analysis of variance with 999 permutations and Bray–Curtis distances measure. This analysis was performed using the function “*adonis2*” from the vegan package in R. We also tested for differences in the homogeneity of multivariate dispersion between the samples using the “*betadisper*” function in the vegan package. The measured physico-chemical factors (aka environmental factors) were checked for collinearity using a Spearman rank correlation test. The *P*-values were adjusted with the Bonferroni correction method. Highly correlated factors were removed from the dataset (Fig. S3). Furthermore, to identify the subset of environmental factors that best predicted the patterns in the microbial community structures and their relationship with the microbial communities, we conducted a BIO-ENV analysis. For this analysis we used the Bray–Curtis dissimilarity matrix of the microbial taxa and the standardized Euclidean distance matrix of the environmental data using the “*bioenv*” function from the vegan package. To assess potential interactions between phytoplankton and the putative parasites, we performed correlation analysis with infected phytoplankton biomass and ASV abundance of the parasite groups that were identified by microscopy to be responsible for the infections, using the nonparametric Kendall’s tau correlation test. The relationship between parasite abundance (expressed as infected host biomass) and total host biomass (infected + uninfected biomass) was analyzed using the nonparametric Spearman’s rank correlation test.

## Results

### Physico-chemical characteristics of sampling sites during the study period

The sampling sites differed with respect to environmental conditions (Supplementary file S2). The semienclosed inner coastal sites (INNER) had lower salinity (range: 2.3–3.9), lower inorganic nutrients (PO_4_ range: 3.3–10 µg l^−1^, NH_4_ range 2.4–3.4 µg l^−1^) and lower Chl *a* (range: 0.3–2.3 µg l^−1^) compared to the more exposed outer archipelago sites (OUTER), which had higher salinity (range: 4.8–5.5), higher inorganic nutrients concentrations (PO_4_ range: 12.7–39.1 µg l^−1^, NH_4_ range 2.1–92.5 µg l^−1^), and higher Chl *a* (range: 1.2–4.4 µg l^−1^). Surface water temperature fluctuated during the sampling period (range; 20.6–28.1°C) with highest temperatures recorded on 27 July. The average temperature was slightly higher in INNER sites (24.5°C ± 2) compared to OUTER sites (23°C ± 2).

### Diversity and composition of eukaryotic and fungal communities in the water column and sediment

**Figure 1.**
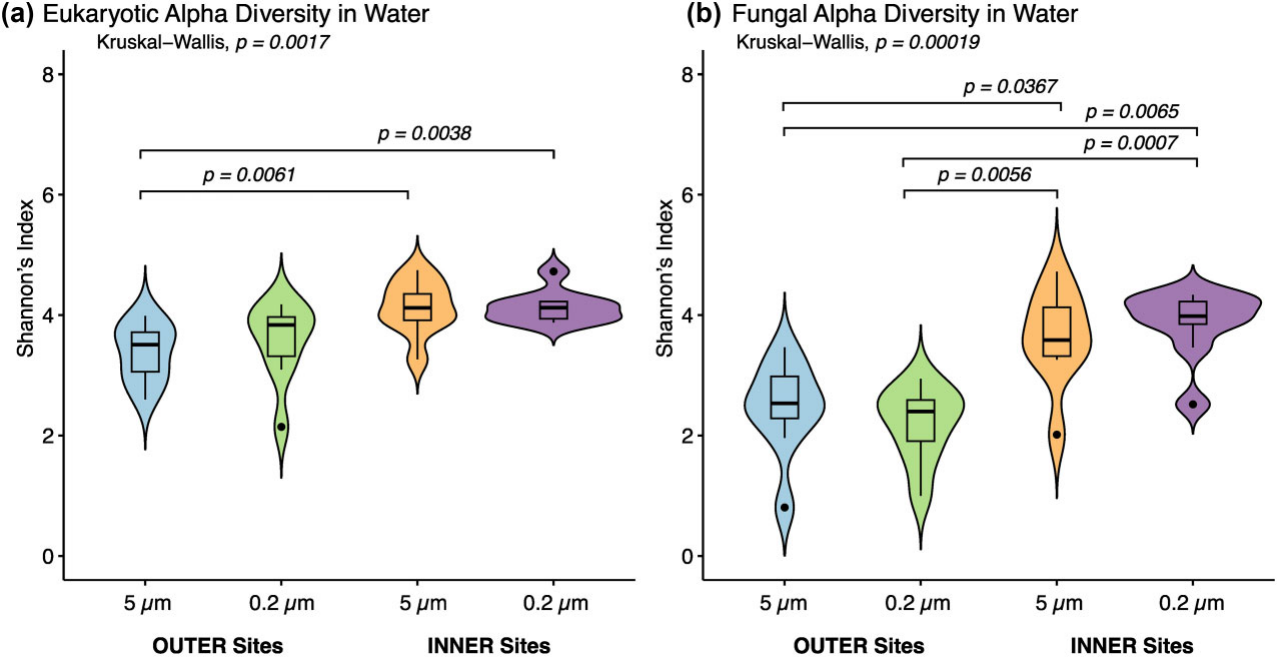
Diversity of the eukaryotic (a) and fungal (b) communities in the different sampling sites (outer and inner sites) and size fractions (5 µm = >5 µm, 0.2 µm = <5 µm). Significant differences are represented by * (0.05), ** (0.01).

In general, eukaryotic alpha diversity was significantly higher in INNER sites compared to OUTER archipelago sites (*P* = .00017). This trend was particularly evident in the larger size fraction (>5 µm), where INNER sites exhibited significantly higher (4.13 _Shannon index avg_,) eukaryotic alpha diversity (*P* = .0061) than OUTER sites (3.37 _Shannon index avg_,) (Fig. 1a). Conversely, no significant difference was observed in the smaller size fraction (<5 µm) between INNER and OUTER sites (*P* = .0758). Similarly, fungal alpha diversity was significantly higher in INNER sites (*P* < .01) compared to OUTER sites in both the smaller size fraction (<5 µm) (*P* = .007) and the larger size fraction (>5 µm) (*P* = .0367) (Fig. 1b).

**Figure 2.**
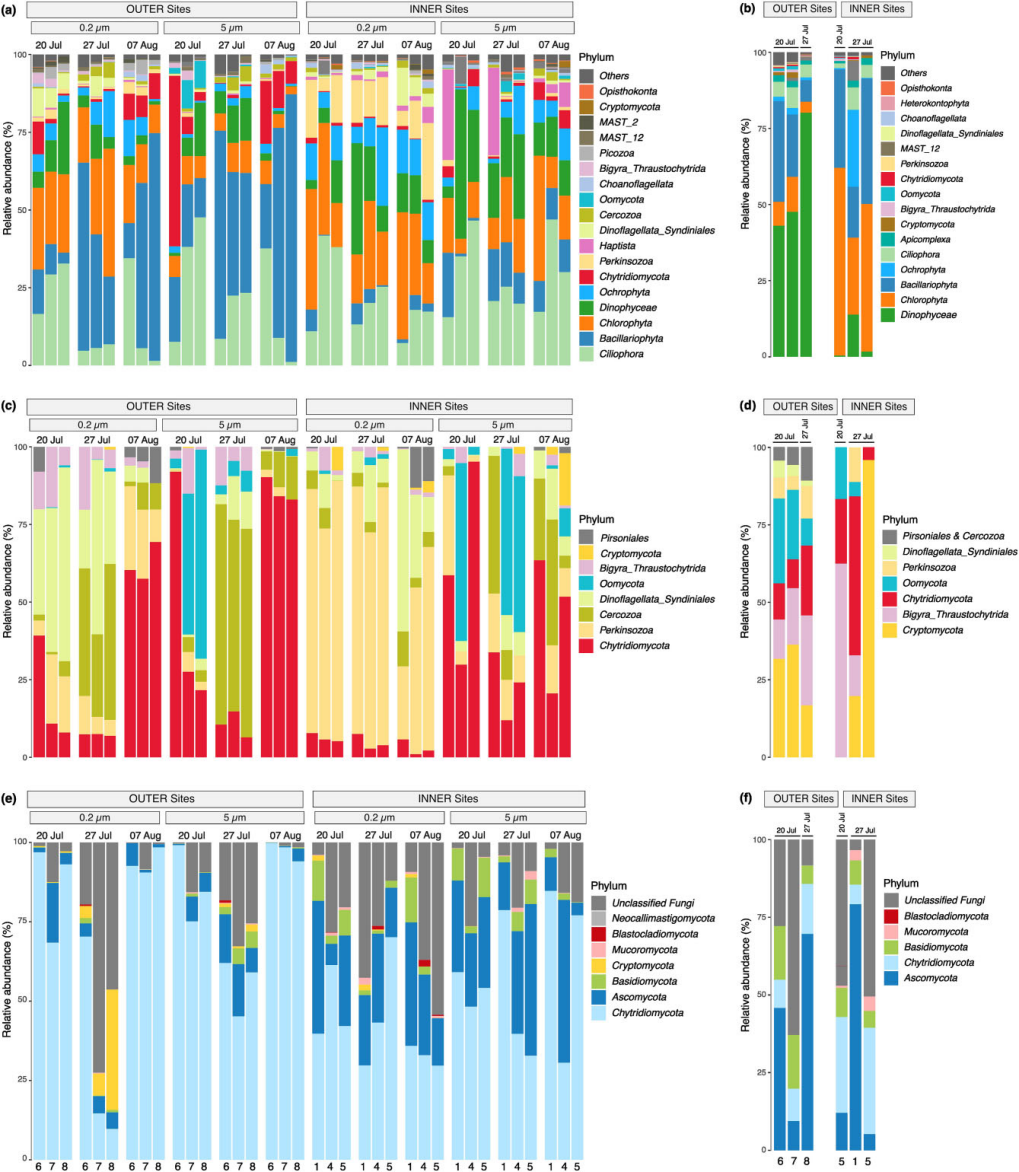
Community composition of eukaryotes, putative parasite taxa, and fungi in water (a, c, and e) and sediment (b, d, and f) for the different size fractions at the different sampling dates and sites (outer and inner sites). The *x*-axis depicts the individual sampling sites.

*Eukaryotes—*among the main phytoplankton taxa, diatoms were on average relatively more abundant in water samples at OUTER sites (37% ± 25) compared to INNER sites (7% ± 7). In contrast, Chlorophyta and Dinophyceae were relatively more abundant in INNER sites (21% ± 11 and 16% ± 12) compared to OUTER sites (14% ± 10 and 7% ± 6) (Fig. 2a). Microscopy-based phytoplankton analysis showed a similar abundance pattern for these main phytoplankton groups (Fig. S4). Overall, phytoplankton biomass was higher at OUTER sites (2713 µg l^−1^ ± 1372) compared to INNER sites (1160 µg l^−1^ ± 1030). Cyanobacteria, which were not covered by the metabarcoding analysis, accounted for the highest biomass in the phytoplankton community in all OUTER and most INNER sites (Fig. S4). OUTER sediment samples were dominated by Dinophyceae (57% ± 20), whereas Chlorophytes and Bacillariophyta dominated in INNER sites representing 45% (± 18) and 30% (± 12), respectively, of the eukaryotic community (Fig. 2b).

Taxonomic groups known to include phytoplankton–parasite species (Table S1) were present in all sites in the water (Fig. 2a and c), except for Aphelidiomycota which was only present in INNER sites, and at a very low relative abundance (<1% of ASVs and reads). Collectively, these taxa accounted for 23% and 20% of all eukaryotic ASVs. In the water column, they constituted 16% of the reads at OUTER sites and 12% at INNER sites. Chytridiomycota and Perkinsozoa were proportionally the most abundant groups, with Perkinsozoa being particularly abundant in the smaller size fraction in INNER sites (Fig. 2a and c). The OUTER sites had on average a higher relative abundance of Chytridiomycota (8% ±13; range 0.3%–56%) compared to INNER sites (2% ± 2; range 0.2%–6%) (Fig. 2a). Putative parasite taxa had similar proportions of ASVs in the sediment, however, in much lower relative abundances (<4%) in both OUTER and INNER sites (Fig. 2b). Syndiniales, Pirsonales and Cercozoa were absent in INNER sediment sites. Cryptomycota was proportionally the most abundant putative parasitic group in sediment samples in both OUTER and INNER sites (0.7% ± 0.7; range 0%–2%). (Fig. 2b and d).

*Fungi—*the highest proportion of fungal ASVs and reads belonged to Chytridiomycota and Ascomycota in both sediment and water at all sites (Fig. 2e and f). Chytridiomycota were on average the most abundant group in the water in both OUTER (75% ± 28; range 10%–99%) and INNER (50% ± 18; range 30%–85%) sites. Ascomycota were relatively more abundant in INNER water (26% ± 13; range 4%–51%) compared to OUTER water (6% ± 6; range 0.1%–18%) (Fig. 2e). Sediments were more variable, with on average higher proportions of Ascomycota (37% ± 33; range 5%–80%) compared to Chytridiomycota (18% ± 12; range 6%–34%) (Fig. 2f).

### Community structure of eukaryotes and fungi

**Figure 3.**
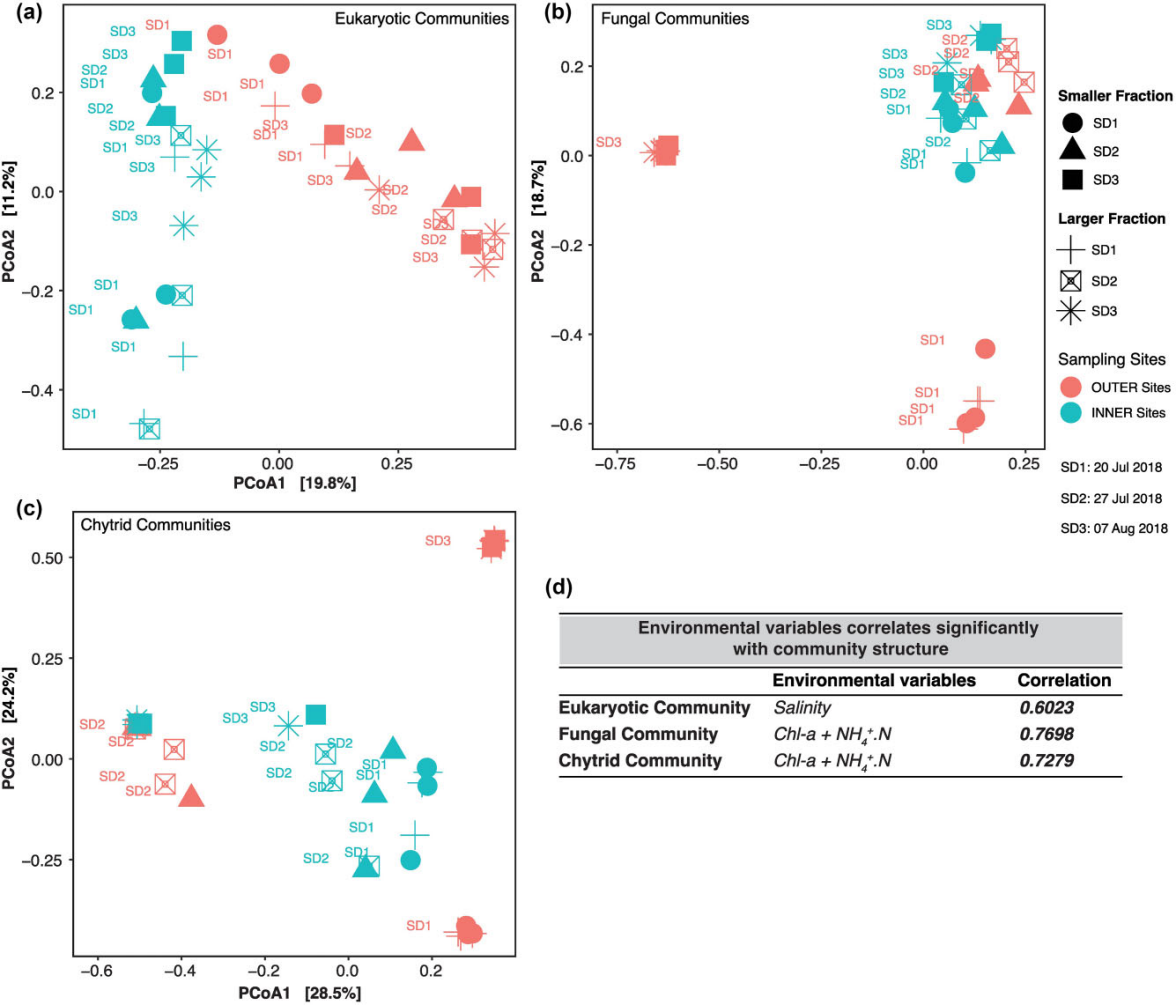
PCoA of eukaryotic (a), fungal (b), and chytrid (c) community for the different size fractions, sampling sites, and sampling dates. The environmental variables which most effectively explained variation in community structure were salinity for eukaryotes, and Chl-*a* and NH_4_-N for fungi and chytrids (d). SD = sampling date.

*Eukaryotic community*: the PCoA analysis revealed a distinct separation of eukaryotic communities along two axes, where the first axis explained 19.8% and the second axis 11.2% of the observed variation (Fig. 3a). Significant differences were observed between OUTER and INNER sites (*F_(site)_* = 9.87, *P* = .001). Moreover, both size fraction (*F_(size fraction)_* = 4.73, *P* = .001) and sampling date (*F_(sampling date)_* = 3.85, *P* = .001) significantly influenced the eukaryotic community structure. Notably, these effects were dependent upon the sampling site, as evidenced by significant interactions (*F_(site x sampling date)_* = 3.17, *P* = .001; *F_(site x size fraction)_* = 1.88, *P* = .001) (Table S2a). Furthermore, the variation in the community structure within OUTER and INNER sites was explained more by sampling date (OUTER: *R^2^*= 0.42, *P* = .001, INNER: *R^2^*= 0.21, *P* = .002) than the size fraction (OUTER: *R^2^*= 0.12, *P* = .003, INNER: *R^2^*= 0.16, *P* = .001).

The relationship between eukaryotic community structure and environmental variables (salinity, temperature, pH, Chlorophyll-a, Si, Total-P, Total-N, and NH4^+^-N) was determined by BioEnv analysis and we found that the salinity alone best predicted the eukaryotic community structure (BioEnv correlation = 0.602) (Fig. 3d, Table S3a).

*Fungal community*: the PCoA ordination of the fungal community displayed differentiation between samples from OUTER and INNER sites, with two axes explaining 41.2% of the total variation (Fig. 3b). We found that sampling site (*F_(site)_*= 11.38, *P* = .001), sampling date (*F_(sampling date)_*= 10.64, *P* = .001), and the size fraction (*F_(size fraction)_*= 2.41, *P* = .017) significantly influenced the structuring of the fungal communities. The effect of sampling date on the community structure was dependent on the site (*F_(site x sampling date)_* = 9.84, *P* = .001), with a higher effect size observed in OUTER sites (*R^2^*= 0.80) compared to INNER sites (*R^2^*= 0.33) (Table S2b). Furthermore, we found that the interplay between Chl *a* and NH_4_-N was most effective in explaining variations in fungal community structure (BioEnv correlation = 0.77) (Fig. 3d, Table S3b). Similar results were obtained when analyzing the chytrid community separately, except that the size fraction did not influence the chytrid community structure (Fig. 3c and d, Tables S2c and S3c).

### Detection and prevalence of phytoplankton parasites

**Figure 4.**
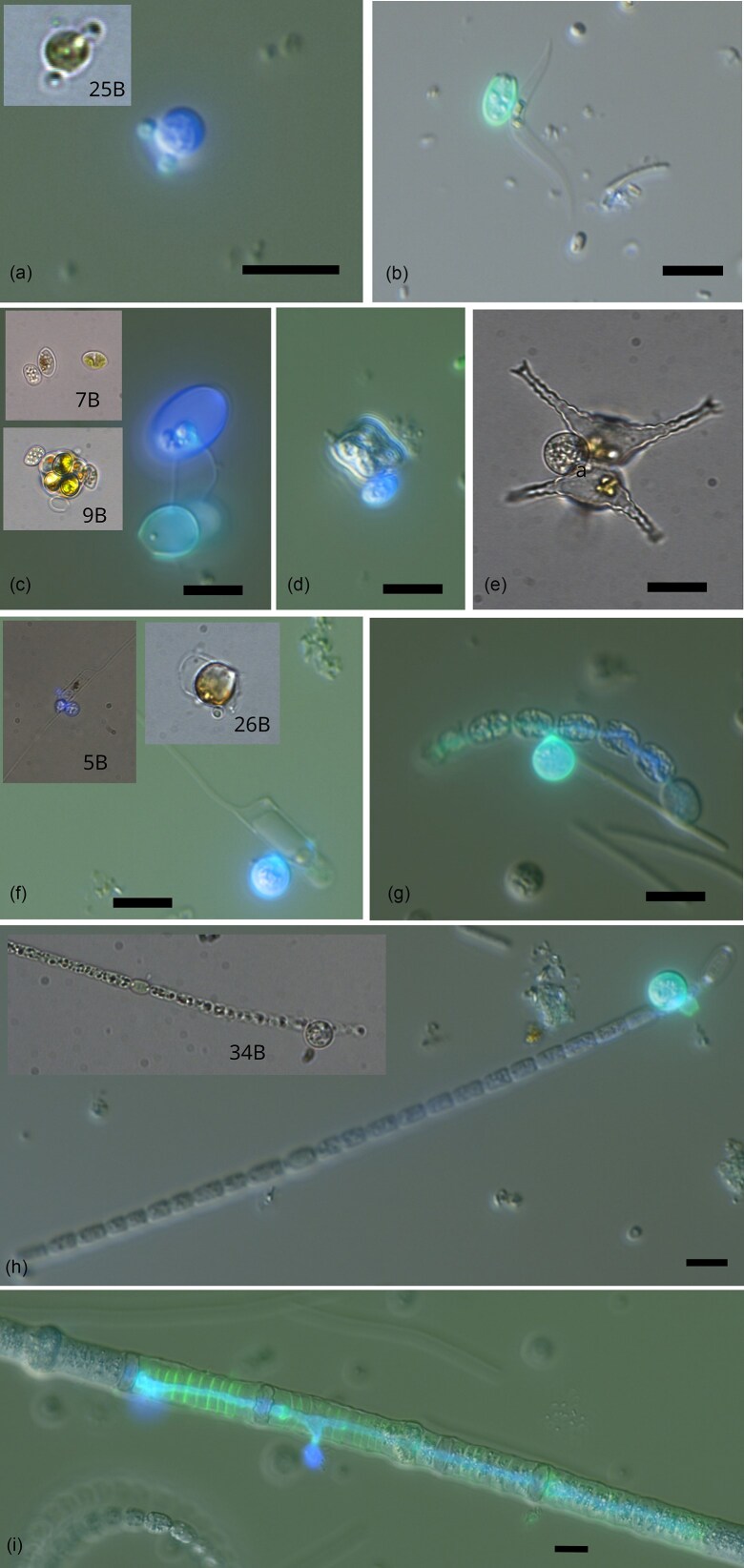
Overlay of transmitted light and fluorescence microscopy images of the observed parasite-infected phytoplankton species. Chytrid infections on unidentified green unicellular sp. (a), *M. contortum* (b), *Oocystis* sp. single cell and colonial (c), *T. minutum* (d), *Staurastrum* sp. (e), *C. minimus* (f), *Dolichospermum* sp. (g), and *Aphanizomenon* sp. (h). Oomycete infection on *Nodularia* sp. (i). Small images depict single cell isolates. Parasites are stained with CFW (387/11 nm excitation and 442/46 nm emission) and Wheat Germ Agglutinine (482/35 nm excitation and 536/40 nm emission). Scale bar = 10 µm.

Microscopy analysis detected parasite infections in 10 out of a total of 65 phytoplankton taxa, including two diatoms, five green algae, and three cyanobacteria (Fig. 4, Table 1). Eight host taxa were present in both OUTER and INNER sites, wherefrom three host taxa were infected in both OUTER and INNER sites [small (<5 µm) unidentified green unicellular sp., *Oocystis* sp. (single cell and colonial), and *Aphanizomenon* sp.] and five host taxa were only infected in either INNER sites (*Chaetoceros minimus, Chaetoceros* sp.) or OUTER sites (*Monoraphidium contortum, Dolichospermum* sp., and *Nodularia* sp.). *Tetraedron minutum* and *Staurastrum* sp. were only present (and infected) in INNER sites. The morphological features of all parasites corresponded to chytrids, except for the parasite infection on *Nodularia* sp., which displayed a phenotype consistent with an oomycete (Fig. 4h).

**Table 1.** Summary table of phytoplankton–parasite interactions observed in this study. n.a. = not applicable, n.o. = not observed.

Infected host species	Single cell parasite ID	rRNA genes	Parasite phylogeny	ASV relative abundance* (range)		Prevalence of infection (range)	
				INNER	OUTER	INNER	OUTER
Green unicellular sp. (<5 µm) (Chlorophyta)	25B	Partial SSU Partial LSU	Zygophlyctidiales (Chytridiomycota)	0%–2%	0%–80%	0%–14%	0%–7%
*Oocystis* sp. (single cell) (Chlorophyta)	7B	Complete 18S	Zygophlyctidiales (Chytridiomycota)	0%–72%	0%–51%	0%–35%** Oocystsis spp.	0%–56%** Oocystsis spp.
*Oocystis* sp. (colony), (Chlorophyta)	9B	Complete 18S	Zygophlyctidiales (Chytridiomycota)	0%–11%	0		
*Chaetoceros minutum*	5B	Complete 18S	Rhizophydiales (Chytridiomycota)	0%–30%	0	0%–90%	0
*Chaetoceros* sp. (Bacillariophyceae)	26B	Partial LSU				n.o.	n.o.
*Aphanizomenon* sp. (Cyanophyta)	34B	Partial LSU	Rhizophydiales (Chytridiomycota)	0%–4%	0%–10%	0%–1.5%	0%–1%
*Staurastrum* sp. (Chlorophyta)	20B 21B	Complete 18S	Rhizophydiales (Chytridiomycota)	0%–1%***	0***	n.o.	n.o.
*Monoraphidium contortum* (Chlorophyta)	n.a.		n.a.	n.a.	n.a.	0%	0%–60%
*Tetraedron minutum* (Chlorophyta)	n.a.		n.a.	n.a.	n.a.	0%–15%	n.a.
*Dolichospermum* sp. (Cyanophyta)	n.a.		n.a.	n.a.	n.a.	0%	0%–1.5%
*Nodularia* sp. (Cyanophyta)	n.a.		Oomycota	n.a.	n.a.	0%	0%–85%

* Relative abundance of the corresponding ASV (>99% sequence similarity) within the fungal community in water samples. ** Due to lugol fixation breaking up *Oocystis* colonies in single cells, we could not accurately quantify single celled and colonial *Oocystis* sp. separately. Prevalence of infection is therefore based on the combined population of *Oocystis* sp. *** Corresponding ASV had 98% sequence similarity with 20B and 21B.

**Figure 5.**
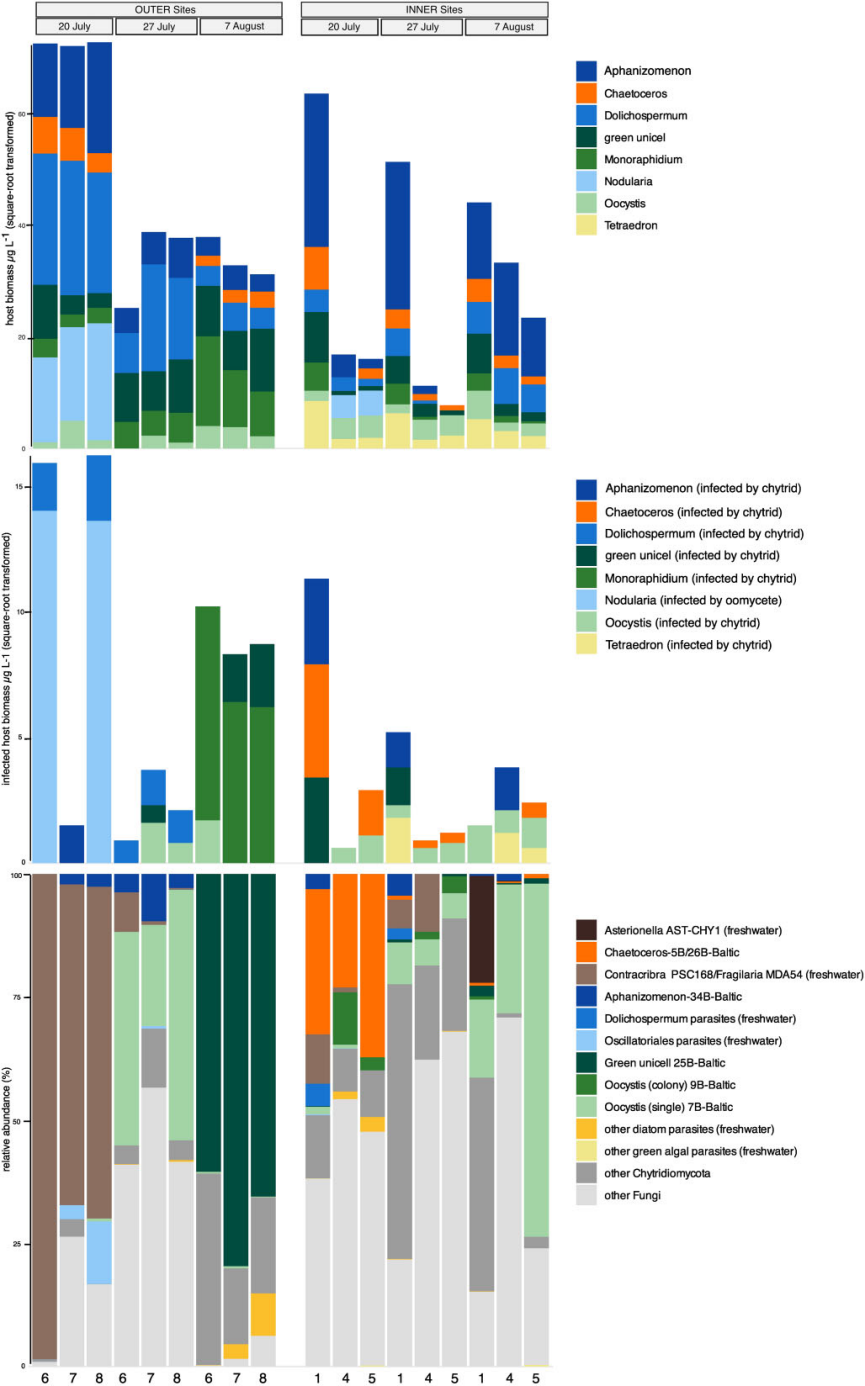
Microscopy-derived estimates of total host biomass (a), infected host biomass by chytrids and oomycetes (b), and relative abundance (c) of identified phytoplankton–parasite chytrids within the total fungal community (>99% sequence similarity with ASVs). The *x*-axis depicts the individual sampling sites.

Highest prevalence of infection was found on *C. minimus* (90% INNER-5), followed by *Nodularia* spp. (85% OUTER-6), *M. contortum* (60% OUTER-8), *Oocystis* spp. (56% OUTER-8), *T. minutum* (15% INNER-4), green unicellular sp. (14% INNER-1), *Dolichospermum* spp. (1.5% OUTER-6,8), and *Aphanizomenon* spp. (1.5% INNER-1) (Table 1). Parasite infections on *Staurastrum* sp. and *Chaetoceros* sp. were only detected in live samples after several days of incubation. In case of *Staurastrum*, the host was not present in lugol samples and thus prevalence of infection could not be quantified. The average percentage of total infected phytoplankton biomass was 0.5% (range 0.07%–1.4%) in INNER sites and 2.4% (range 0.04%–5.8%) in OUTER sites. When excluding the oomycete infection on *Nodularia* sp. and only taking chytrid infections into account, the average infected biomass percentage in OUTER sites dropped to 1.4%. Infected host biomass generally followed the host biomass (Fig. 5a and b) with an overall significant positive correlation (Spearman’s rho = 0.63, *P* = .006) across all sites and species. Notable outliers were *Nodularia, Aphanizomenon*, and *Dolichospermum*, which had consistent biomass across the three OUTER sites on the first sampling date but displayed variable infection rates. *Nodularia* and *Dolichospermum* infections were only present at OUTER-6 and OUTER-8, while *Aphanizomenon* infections were only observed at OUTER-7 (Fig. 5a and b). *Chaetoceros minimus* had similar biomass levels at OUTER and INNER sites, but was only infected at INNER sites (Fig. 5a and b). Across the three sampling dates, we observed short-term temporal variability in host–parasite interactions at both INNER and OUTER sites (Fig. 5b). Initially, OUTER sites showed a higher prevalence of cyanobacteria infections, whereas INNER sites displayed a greater incidence of diatom infections during the same period. Subsequent samplings revealed a shift, with infections of green algal parasites becoming more prominent across both sites. This temporal shift was most distinct at the OUTER sites, where initial dominance of cyanobacterial host species led to the dominance of cyanobacteria parasites. These were completely replaced by green algal parasites infecting green unicellular sp. and *M. contortum* on the last sampling date, when these species dominated the host community.

**Figure 6.**
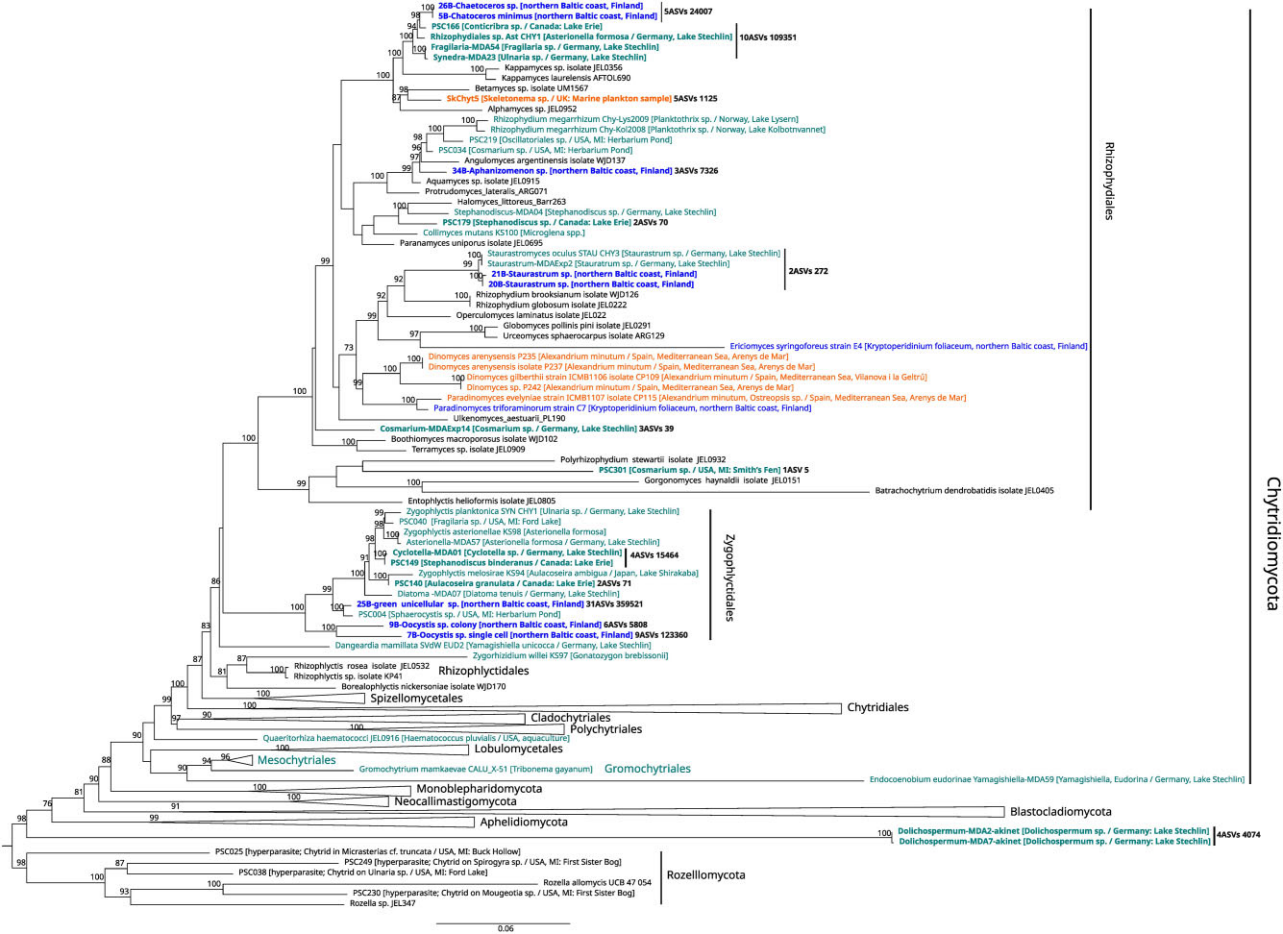
Maximum-likelihood phylogenetic tree of full-length rRNA and 28S rRNA sequences, including Rozellomycota, Aphelidiomycota, Blastocladiomycota, Neocallimastigomycota, Monoblepharidomycota, and representatives from most orders within Chytridiomycota. New chytrid sequences from single-cell isolates from brackish coastal sites generated in this study are labeled in bold dark blue. Published sequences from phytoplankton parasites are color-coded according to their habitat of isolation; cyanine blue for freshwater, dark blue for brackish, and orange for marine. Parasite sequences that were identified in the metabarcoding dataset (>99 sequence similarity with an ASV) are highlighted in bold. For all identified parasite sequences, the number of corresponding ASVs and the reads obtained for the ASVs are provided. Values in the nodes represent bootstrap (%) statistical support, with only values >70% shown.

We manually isolated 31 infected single cells/colonies [11 *Oocystis* sp. (single cell and colony form); 5 *Aphanizomenon* sp.; 3 *Dolichospermum* sp.; 3 *C. minimus*; 3 *Chaetoceros sp*.; 3 *Staurastrum* sp.; 2 *M. contortum*; and 1 green unicellular sp.] for single cell sequencing. From these, we obtained five complete FRO sequences, three partial LSU rRNA sequences, and one partial SSU rRNA sequence from a total of eight infected cells/colonies, including *Chaetoceros* spp., *Oocystis* sp. (single cell and colony form), green unicellular sp., *Staurastrum* sp., and *Aphanizomenon* sp. (Table 1). All sequences belonged to Chytridiomycota and their phylogenetic position is shown in Fig. 6. The two chytrid sequences derived from *Staurastrum* sp. were identical and had 98% full-length rRNA sequence similarity with *Staurastromyces oculus*, a chytrid species infecting the desmid *Staurastrum* sp. in freshwater lakes. Chytrid isolates infecting *C. minimus* (5B) and *Chaetoceros* sp. (26B) had identical LSU rRNA sequences and clustered together with diatom-associated parasites isolated from freshwater (Rhizophydiales clade RH- *sensu*; Van den Wyngaert et al. 2022, Seto et al. 2023). The nearly full length rRNA sequence of 5B-*Chaetoceros* showed 98% similarity with Rhizophydiales sp. isolate PSC166 (OQ702890), a parasitic chytrid on the centric diatom *Conticribra* sp. Chytrid isolate 34B infecting *Aphanizomenon* sp. branched sister to an endoparasitic chytrid of the desmid *Cosmarium* sp. (OQ702854), with which it showed 97% LSU rRNA sequence similarity. Chytrid 25B, isolated from a small (5 µm) unicellular green algal species, and Chytrids 7B and 9B, isolated from a single-celled and colonial *Oocystis* sp., respectively, formed two distinct clades within the order Zygophlyctidales. Isolate 25B-green unicellular sp. showed a close affiliation (97% LSU rRNA sequence similarity) to a parasitic chytrid isolated from the freshwater green alga *Sphaerocystis* sp. (OQ702842) (Fig. 6).

All chytrid single-cell isolates were detected (i.e. ASVs with >99% sequence similarity) in water samples in both the eukaryotic and fungal amplicon dataset, except for 20B/21B-*Staurastrum*, for which sequences showed only 98% similarity to two ASVs in the fungal dataset (Table 1). ASVs from isolates 20B/21B-*Staurastrum*, 5B/26B-*Chaetoceros*, and 9B-*Oocystis* (colony) were only detected in INNER sites, despite the presence of the host species *Chaetoceros* spp. and *Oocystis* sp. in both sites. The other isolates; 7B-*Oocystis* (single cell), 25B-green unicellular, and 34B-*Aphanizomenon*, were present in both INNER and OUTER sites (Table 1). Together they represented 30% of total fungal reads, with ASVs matching 25B-green unicell (ASV1 and ASV2) being the most abundant (21% of total fungal reads) (Fig. 5c, Table 1). The next most abundant fungal ASVs (ASV3 and ASV4) matched both chytrids isolated from freshwater and infecting diatoms *Fragilaria* sp. and *Conticribra* sp. with 99% similarity, and showed 98% similarity to *Chaetoceros*-5B. These ASVs reached high relative abundances up to 90% at OUTER-6 (Fig. 5c). ASVs matching with chytrid parasites of green algae and the cyanobacterium *Dolichospermum* spp. isolated from freshwater, were only detected in low relative abundance at INNER sites. Fungal ASVs matching with >99% similarity to a sequence of the marine chytrid parasite SkChyt5, isolated from the diatom *Skeletonema* sp., were detected once in a water sample at a very low relative abundance (0.05%, not visible in Fig. 5c) in OUTER-7. Overall, 55% of the total fungal reads in the water samples corresponded to parasitic chytrids of phytoplankton.

In contrast to the microscopy-based observations, the molecular data indicated that diatom-associated chytrid parasites were most prevalent at the initial sampling date in both OUTER and INNER sites. In agreement with microscopy, green algal parasites became also more prominent in subsequent samplings. At the OUTER sites in particular, shifts in the dominant parasite taxa were observed, with initial detection of diatom parasites followed by increased representation of ASVs affiliated with Oocystis (7B-Oocystis) and green unicellular algae (25B-green-unicell) by the final sampling date (Fig. 5b and c).

There was an overall tendency of higher chytrid ASV richness in sediments (47 ± 39; range 7–101) compared to water (20 ± 16; range 4–58). However, the relative abundance of identified parasitic chytrids was much lower in sediment samples compared to water samples, representing only about 1% of the fungal reads. Some parasites were exclusively detected at INNER sediment sites (25B-green unicellular) or OUTER sediment sites (34B-*Aphanizomenon*), even though they were present in the water samples at both sites. The highest relative abundance of 5B-*Chaetoceros* in sediment occurred at INNER-5, reaching 1%, where it was also more abundant in the water column (37%). The ASVs associated with the *Skeletonema* parasite SkChyt5 were the only instance where its relative abundance was higher in the sediment (up to 2% at INNER-5) compared to the water column, where it was absent at INNER-5 and had a maximum abundance of 0.05% at OUTER-7.

Some nonfungal eukaryotic phytoplankton parasites were only detected in the sequence data. ASVs matching with *Cryothecomonas* (max 1% relative abundance) and *Pirsonia* (max 4% relative abundance), which are known to parasitize marine diatoms, occurred in INNER and OUTER water sites. *Amoebophrya* (max 12% relative abundance), a parasite of marine dinoflagellates, was mainly present in the smaller size fraction in both INNER and OUTER sites. *Aplanochytrium*, known to feed on living marine diatoms, was detected only in sediment samples at very low relative abundance (max. 1% relative abundance). Perkinsozoa showed high relative abundances in the smaller size fraction in INNER sites. *Parvilucifera*, a known dinoflagellate-parasite genus, was present in very low abundance (<0.01% relative abundance).

### Phytoplankton–parasite interactions

**Figure 7.**
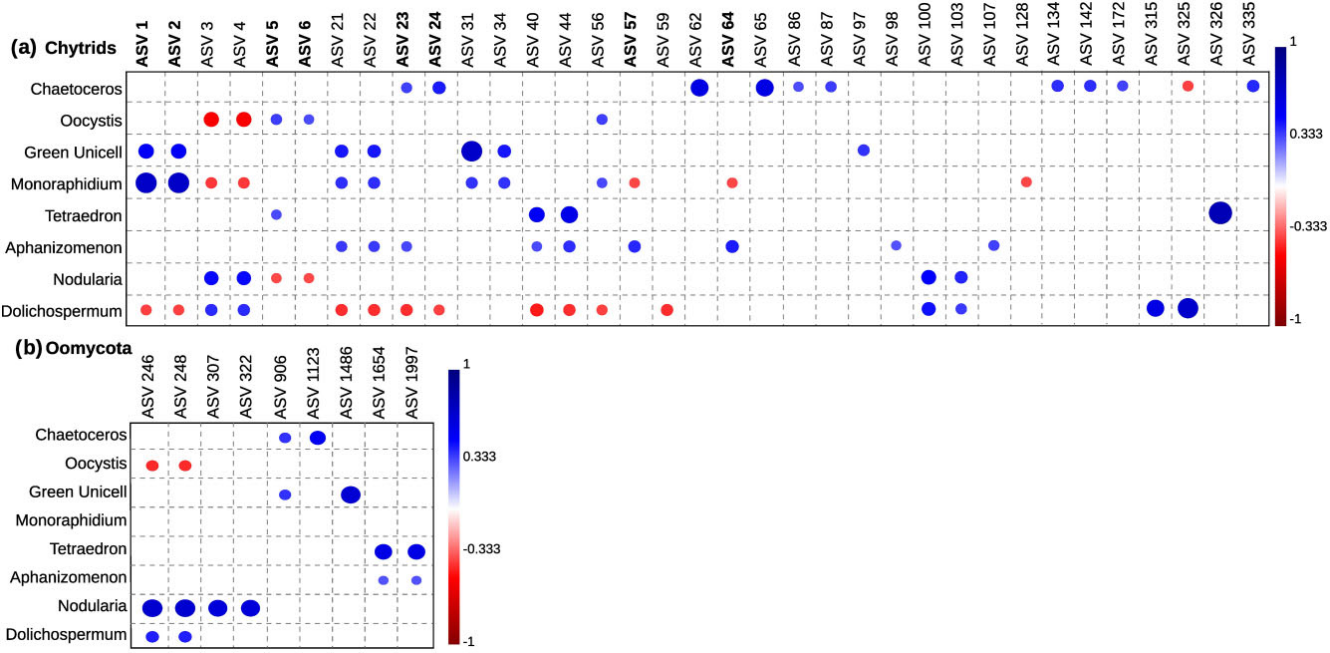
Correlation between infected phytoplankton host species biomass and chytrid ASVs (a), and oomycota ASVs (b).

Empirically observed phytoplankton–parasite interactions were validated by a positive correlation (significant for uncorrected *P*-values) between the relative abundance of the ASVs corresponding to the single-cell chytrid parasite isolates obtained in this study and the infected biomass of their respective host species (Fig. 7). Some of these chytrid parasite ASVs showed also a positive correlation with other infected host species (Fig. 7a). For example, ASV5, associated with *Oocystis* sp. (7B) was also positively correlated with infected *Tetraedron* biomass. All ASVs (ASV1 and ASV2) associated with green unicellular species (25B) were positively correlated with infected *Monoraphidium* biomass, and one of the ASVs (ASV23) associated with *Chaetoceros* (5B) was positively correlated with infected *Aphanizomenon* biomass. Infected biomass of *Dolichospermum* and *Nodularia* correlated with ASVs (ASV100 and ASV103) corresponding to an uncultured chytrid parasite isolate of the cyanobacteria *Oscillatoriales* sp. (OQ702913). Infected *Tetraedron* biomass and infected green unicell biomass also correlated with ASVs that had 98% (ASV40 and ASV44) and 99% (ASV21 and ASV22) sequence similarity, respectively, with an uncultured chytrid parasite isolate on the putative desmid species *Coelastrum* sp. (OQ702913).

In addition to the identified parasite ASVs, there were other nonidentified ASVs that showed a positive relationship with infected host species biomass. The number of positively correlated ASVs per host species ranged from 3 (*Oocystis*) to 10 (*Chaetoceros*) (Fig. 7a). Infected *Nodularia* biomass showed the strongest positive correlation (significant for corrected *P*-values) with the two most abundant Oomycota ASVs (Fig. 7b; ASV 246 and 248). A blast search against the INSDC nucleotide database of these ASVs gave highest sequence similarity (96%) with freshwater diatom parasite species of the genus *Lagena* (Thines and Buaya 2022).

## Discussion

This study provides the first qualitative and quantitative data on zoosporic parasites infecting phytoplankton at a community level in the Baltic Sea integrating next-generation sequencing with empirical observations. Chytrids were confirmed as key parasites, infecting diverse phytoplankton taxa in brackish coastal environments, with host range and infection prevalence comparable to those observed in freshwater systems (Rasconi et al. 2012, Gsell et al. 2022, Van den Wyngaert et al. 2022).

The highest proportion of chytrid-infected phytoplankton biomass was 1.4% at INNER sites (average 0.5%) and 5.8% (average 1.4%) at OUTER sites. When including oomycete infections on *Nodularia*, it reached an average of 2.3% in OUTER sites. While these values are slightly lower than the average of 2.8% reported from the eutrophic freshwater shallow Lake Müggelsee (Germany) covering a 12-year dataset (Gsell et al. 2022), they underscore the significance of phytoplankton parasitism in brackish ecosystems. Comparable infection rates have also been reported in marine systems. For example, chytrid infections in Arctic sea-ice diatom communities reached up to 1% during the peak of the spring bloom (Hassett and Gradinger 2016), while chytrid and oomycete infections in benthic diatom communities in tidal flats ranged from 1.6% to 6.3%, averaging 3.7%, with most infections attributed to chytrids (Scholz et al. 2016). Metabarcoding also supported the quantitative importance of fungal parasitism on phytoplankton, identifying it as the dominant lifestyle within the planktonic fungal community at multiple sites and occasions over the study period.

Our observation that bloom-forming toxic filamentous cyanobacteria species in the Baltic Sea—A*phanizomenon, Dolichospermum*, and *Nodularia—*are all susceptible to infections by zoosporic parasites is noteworthy, given the increasing frequency of harmful cyanobacterial blooms in the region (Suikkanen et al. 2007, Funkey et al. 2014, Olofsson et al. 2020). Chytrid infections on *Aphanizomenon* and *Dolichospermum* also occur in freshwater lakes (Gsell et al. 2022, Van den Wyngaert et al. 2022), typically exhibiting similar infection prevalence (∼1%–2%) to that observed in this study. However, occasional high infection rates (<90%) have been reported, such as in eutrophic lake Aydat, France (Rasconi et al. 2012, Gerphagnon et al. 2017). Variability in infection rates on cyanobacteria blooms has been linked to toxins as a defense mechanism (Rohrlack 2013, Weisbrod et al. 2020) and to parasitism’s impact on akinetes (overwintering resting cells), which may reduce host recruitment and hinder the success of other chytrids reliant on vegetative blooms (Gerphagnon et al. 2017). An unidentified oomycete infection in *Nodularia*, with prevalence reaching up to 85% of the cyanobacterial population, underscores the hidden diversity of zoosporic parasites and their potential to exert significant top-down pressure on toxic cyanobacteria blooms in the Baltic Sea. Further research is imperative to better understand cyanobacteria–parasite interactions and integrate their role in driving cyanobacteria bloom dynamics, bloom toxicity, and nutrient cycling (Munkes et al. 2021).

Heterogeneous environmental conditions at the OUTER and INNER sites led to distinct microbial communities and phytoplankton–parasite interactions, with salinity as one of the primary influencing factors. At the INNER sites, salinity levels approached the critical threshold of 2–3, where freshwater phytoplankton communities typically shift toward an assemblage of brackish-water and marine species (Niemi 1982). The INNER sites exhibited typical freshwater taxa, such as desmids, which were absent in the more saline OUTER sites. Given the high host specificity of chytrids (Canter and Jaworski 1978, Van den Wyngaert et al. 2017, Kagami et al. 2021, Fernández-Valero et al. 2022), it is very likely that the chytrid species infecting desmids, like *Staurastrum* and *Tetraedron*, were consequently restricted to the less saline INNER sites, in accordance with the distribution of their hosts. The cooccurrence of freshwater taxa alongside brackish species and their specific interactions likely contributed to a higher eukaryotic and fungal diversity in the INNER sites (Kisand et al. 2005, Lobo et al. 2024). Salinity has also been identified as the main driver of planktonic fungal communities in the Baltic Sea, with a threshold of 8 (Rojas-Jimenez et al. 2019). In our study, which focused on a smaller spatial scale and salinities below 8, we found that Chl *a* and NH4 explained most fungal community variation. High levels of Chl *a*, as proxy for phytoplankton biomass, likely promote growth of fungal parasites by increasing the availability of potential hosts. The significance of Chl *a* aligns well with our results, indicating that chytrid parasitism on phytoplankton was the dominant lifestyle within the planktonic fungal community.

Infected phytoplankton biomass was, on average, higher in OUTER sites, which exhibited elevated levels of salinity, nutrients, and Chl *a*. This trend likely stems from the increased phytoplankton biomass in these sites, facilitating the transmission and development of parasitic infections (Gsell et al. 2022, Reñé et al. 2023). A significant positive correlation between host biomass and infected biomass across all sites indicates that host abundance significantly influenced the occurrence of parasite interactions, consistent with previous studies (Holfeld 1998, Ibelings et al. 2011, Gsell et al. 2022). However, host abundance alone could not explain the absence of certain host–chytrid interactions. For example, the *Chaetoceros–*chytrid (5B/26B) and *Oocystis*–chytrid (9B) were exclusively detected at INNER sites, despite higher *Chaetoceros* biomass at the OUTER sites. One possible explanation is that physico-chemical conditions in OUTER sites were not conducive to the spread of this chytrid species. Although salinity differences were minimal (2–3 versus 5–6) between OUTER and INNER sites, growth experiments with saprophytic chytrids isolated from low saline (2) coastal sites showed reduced growth at salinity 4, and for some strains, growth was completely inhibited at salinity 8 (Guo et al. 2023). The mechanisms by which chytrids balance osmotic pressure are currently unknown. Alternatively, the *Chaetoceros* population in OUTER sites was potentially resistant to the *Chaetoceros*–chytrid parasite (5B/26B), a mechanism described as variable susceptibility among host–parasite genotypes in lakes (Holfeld 1998, De Bruin et al. 2004). Even over small spatial scales (∼25 km), similar to those in our study, genetically differentiated diatom populations have been found in the Baltic coastal archipelago (Sefbom et al. 2018), suggesting that host–parasite interactions may vary due to ongoing coevolutionary dynamics (Thompson 2005).

Within sites, host–parasite interactions and the metabarcoding data exhibited significant short-term changes of parasitic chytrid species. The small-scale spatial and temporal heterogeneity of plankton communities and their interactions underscores a spatial and temporal patchiness that warrants future investigation with an extended sampling design.

In addition to our single-cell approach, we used cooccurrence analysis to investigate potential phytoplankton–parasite interactions. Although still inferential, this approach uses empirical data of infected host biomass, which aligns more closely with actual host–parasite dynamics. Alongside the specific ASVs identified as chytrid parasites for phytoplankton through our single-cell analysis, our results suggest a broader host range for certain chytrid species. For instance, ASVs of the chytrid (25B) identified as infecting the green unicellular species, showed significant correlations with the biomass of infected *Monoraphidium*. Microscopy confirmed morphological similarities between chytrids infecting both host species, supporting shared host susceptibility. Additionally, ASV 25B emerged as the most abundant fungal ASV, while *Monoraphidium* displayed the highest infected biomass, collectively suggesting that this chytrid may exhibit a generalist parasitic behavior. Positive correlations between infected host biomass and other unidentified ASVs indicate the presence of multiple chytrid parasites affecting the same host population, consistent with previous findings in freshwater lakes (Kagami et al. 2021, Van den Wyngaert et al. 2022).

Applying a similar approach for analyzing the oomycete infection in *Nodularia*, we identified a potential interaction with the two most abundant oomycete ASVs. Manual BLAST analysis linked these ASVs to the genus *Lagena*. Currently, *Lagenidium nodosumon* is the only known oomycete parasite infecting limnic cyanobacteria species like *Lyngbya* sp. (Sparrow 1960). A recent study suggested that *Lagenidium* species, including newly identified parasites infecting freshwater diatoms, may belong to the genus *Lagena* due to similarities in their zoospore production (Thines and Buaya 2022). Isolation and cultivation of the oomycete parasite will be essential to accurately identify the oomycete in question.

The formation of thick-walled resting spores has been observed in the life cycle of several obligate parasitic chytrids (van Donk and Ringelberg 1983, Van den Wyngaert et al. 2017, Seto et al. 2020), likely aiding long-term survival during unfavorable conditions (Doggett and Porter 1996). Therefore, we expected greater diversity of chytrid parasites in the sediment due to the accumulation of these resting spores, forming a parasite seed bank. While we observed higher numbers of chytrid ASVs in sediments, similar to findings in Mediterranean coastal sites (Fernández-Valero et al. 2022, Reñé et al. 2023), we could not confirm that this diversity was associated with a parasitic lifestyle. Parasitic chytrids detected in water were scarce or absent in sediments. Overall, chytrid relative abundance was lower in sediments compared to water, which contained larger proportions of higher and unknown fungi. This contrasts with studies showing Chytridiomycota dominance in sediments (Lobo et al. 2024), which may have limited the detection of low-abundant parasite resting spores. The higher sediment diversity of chytrids likely reflects a mix of different life stages of planktonic chytrids sinking out of the water column and benthic chytrids decomposing organic matter (Lobo et al. 2024) or parasitizing benthic microalgae (Scholz et al. 2016, Fernández-Valero et al. 2023). Although resting spores are presumed to be an important survival strategy for obligate and host-specific parasitic chytrids, there are few empirical observations of resting spores in nature (van Donk and Ringelberg 1983, Doggett and Porter 1996). DNA metabarcoding cannot distinguish between chytrid life stages, leaving the relative abundance of resting spores unresolved. However, a recent study, using a novel DNA extraction method to enrich for lysis resistant fungal structures showed an enrichment of Chytridiomycota in lake sediments of a high-altitude athalassohaline wetland in the Chilean Altiplano (Corona Ramirez et al. 2023). This suggests that thick-walled (lysis-resistant) resting spores may comprise a substantial fraction of the community. Combining molecular-based lysis-resistant enrichment methods with microscopy to directly observe these structures is needed to enhance our understanding of the composition and dynamics of the chytrid seed bank.

Unlike parasitic interactions with phytoplankton that we directly observed for chytrids and an unidentified oomycete, we did not observe any confirmed parasitic interactions involving other taxonomic groups. Perkinsozoa ASVs were abundant at INNER sites, particularly in the smaller (<5 µm) size fraction, but none of the ASVs matched with any of the currently known Perkinsozoa dinoflagellate-parasite species. Although Perkinsozoa parasites have a broad host range, including invertebrates and vertebrates, their ecology and host associations in brackish and freshwater environments remain largely unknown (Jobard et al. 2020). Only recently, the first two Perkinsozoa (*Parvilucifera catillosa* and *Parvilucifera* sp.) and chytrid (*Ericiomyces syringoforeus* and *Paradinomyces triforaminorum*) species have been isolated from a coastal Baltic sea site with salinity 6–7 during a high biomass bloom of the dinoflagellate *Kryptoperidinium triquetrum* (=*K. foliaceum*) (Alacid et al. 2020, Karpov et al. 2021, Reñé et al. 2022). In our field study, dinoflagellates (but not the species *K. foliaceum*) were present in all sites, but did not form high biomass blooms, likely limiting the proliferation of dinoflagellate parasites. The dominance of Perkinsozoa in the smaller <5 µm size fraction likely reflects the presence of motile zoospore stages of species parasitizing larger invertebrates or vertebrates, which were not covered by our sampling. Similarly, ASVs assigned to the parasite genus *Amoebophrya* (Syndiniales), were occasionally abundant in the smaller size fraction at both INNER (max. 12%) and OUTER (max. 9%) sites but only present in very low abundances (<1%) in the larger, phytoplankton-associated size fraction. Currently, confirmed molecular parasitic interactions of *Amoebophrya* (*A. ceratii* species complex) are exclusively with dinoflagellates (Guillou et al. 2023). However, morphology-based descriptions suggest that *Amoebophrya* also infects radiolarians, cnidarians, chaetognaths, and ciliates (Jephcott et al. 2016), though the molecular identity of *Amoebophrya* in these interactions has yet to be confirmed.

The detection of ASVs matching with other nonfungal parasitic genera known to infect diatoms (*Cryothecomonas, Pirsonia*, and *Aplanochytrium*), suggests that, although these taxa are low in relative abundance, their interactions may occasionally play an important role in the Baltic Sea ecosystem. Seasonal and rapid temporal dynamics of specific host–parasite interactions are anticipated to significantly influence the occurrence and relative abundance patterns of parasitic taxa (Käse et al. 2021, Van den Wyngaert et al. 2022, Catlett et al. 2023). To assess the relative importance of the different zoosporic parasite lineages, large-scale analyses based on high-resolution time-series data are imperative (Catlett et al. 2023).

## Conclusion

In conclusion, we demonstrate that parasitic chytrid–phytoplankton interactions are a significant component of microbial communities and dominate the planktonic fungal community during coastal summer phytoplankton blooms in the brackish northern Baltic Sea. Variability in phytoplankton host abundance, species composition, and site-specific conditions shape these interactions, emphasizing the importance of localized studies. The observed small-scale spatial and temporal heterogeneity highlights the need for high-frequency sampling to accurately assess the occurrence and impact of parasites on natural phytoplankton assemblages. The discovery of novel diversity and substantial abundance of parasitic chytrids and oomycetes, particularly in association with harmful cyanobacteria species, highlights their ecological importance. Further research is needed to explore their impact on bloom dynamics and nutrient cycling in the Baltic Sea.

## Supplementary Material

fiaf081_Supplemental_File
