# Supplementary material for: Dynamics of zoosporic parasites in summer phytoplankton communities of the Baltic Sea
Source: FEMS Microbiol Ecol. 2025 Aug 7;101(8):fiaf081. doi: 10.1093/femsec/fiaf081 (PMC12345203; doi:10.1093/femsec/fiaf081)
Supplement: fiaf081_Supplemental_File [file fiaf081_supplemental_file.docx]

**Supplementary Information:** Dynamics of zoosporic parasites in summer phytoplankton communities of the Baltic Sea

Silke Van den Wyngaert, Ali Nawaz, Elisabet Alacid, Steffaney Wood, Albert Reñé, Esther Garcés, Anke Kremp, Christian Wurzbacher

**Material and Methods**

**Figure S1**: Map of field study and sampling sites. The six sampling sites are located in the Tvärminne area, southwest coast of Finland near the Tvärminne Zoological Station (TZS, see smaller insert map). Stations 1,4 and 5 (green color) are located in the shallow, semi-enclosed and sheltered innermost (coastal) zone (Stadsfjärden and the end of Pojoviken fjord), near the village Ekenäs (referred to as INNER sites). Stations 6,7 and 8 (orange) are located in the more exposed outer archipelago zone, around 25 km southwest of the INNER sites at the mouth of the Gulf of Finland, near Tvärminne research station (referred to as OUTER sites). The definition of the zones is based on Niemi (Niemi 1972).


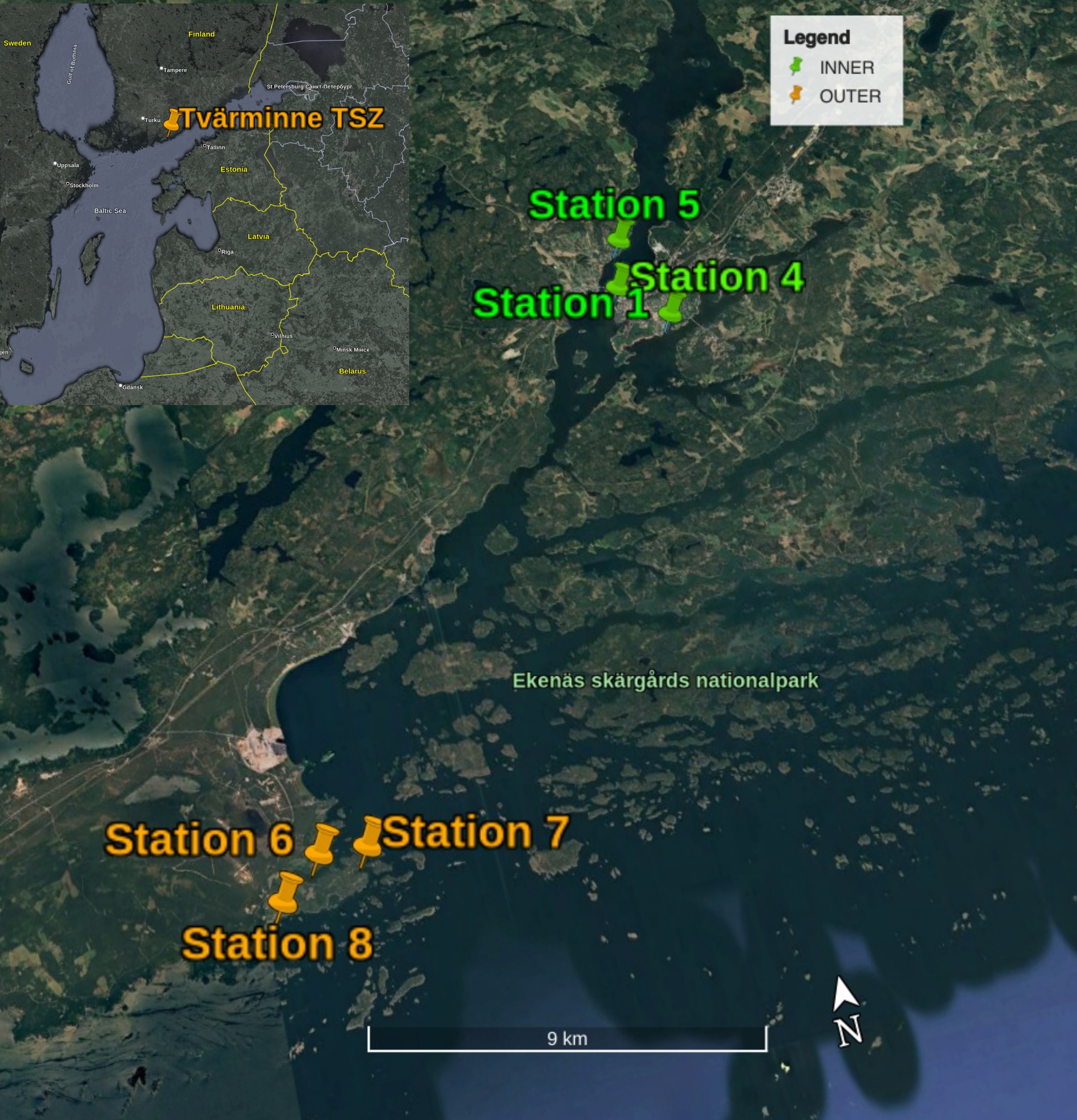


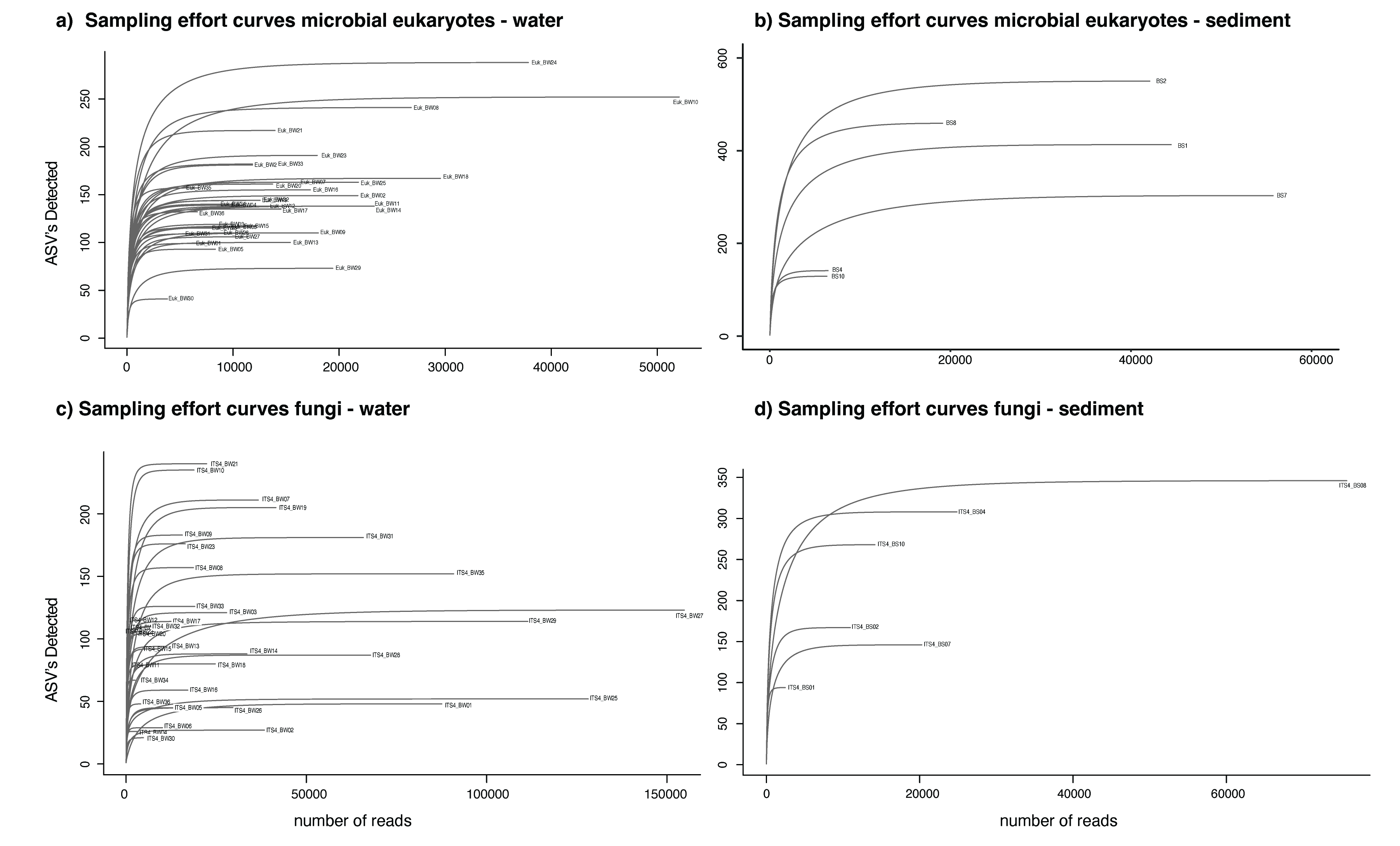


**Figure S2:** Sampling effort curves

**Figure S3**: Heatmap showing the collinearity of the measured physicochemical (environmental) factors, assessed using the Spearman rank correlation test.


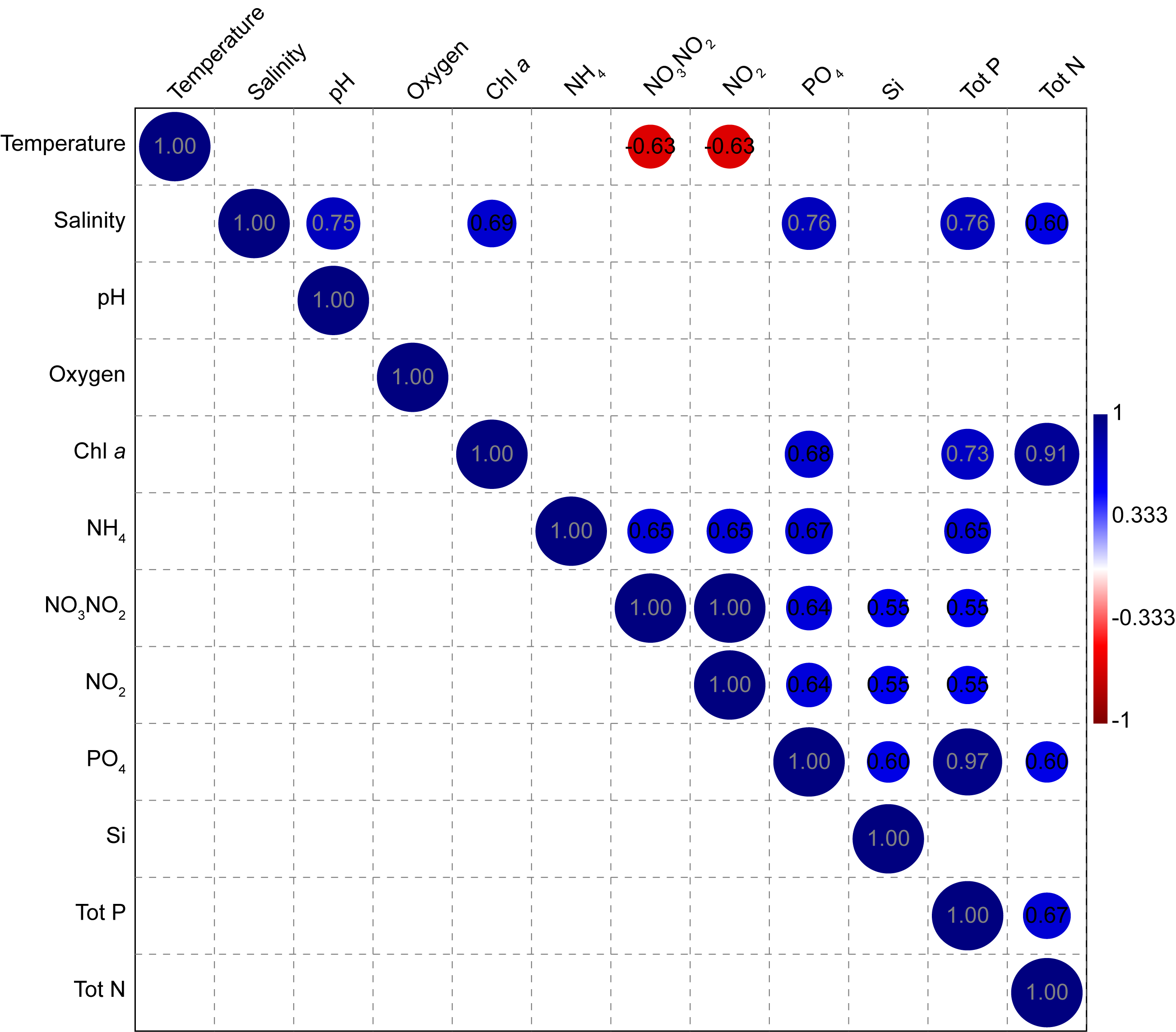


**Results**


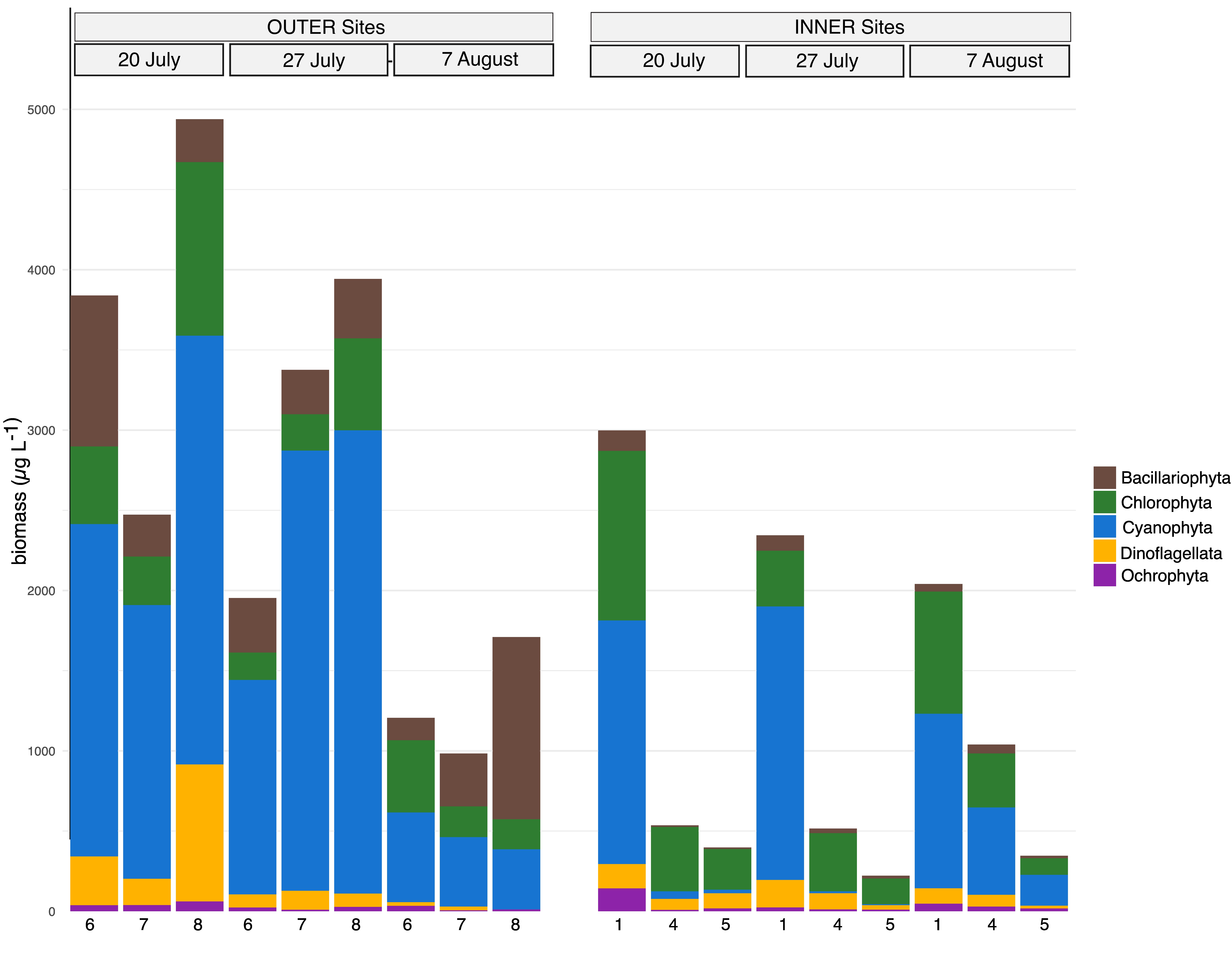
**Figure S4:** Phytoplankton biomass composition based on microscopy analysis

**Table S1:** Eukaryotic lineages which include zoosporic parasite species associated with phytoplankton.

| **Lineages including parasites of phytoplankton** | **reference** |
| --- | --- |
| Chytridiomycota (fungi) | Frenken et al. 2017 |
| Aphelidiomycota (fungi) | Letcher and Powell 2019 |
| Cryptomycota (fungi) | Kagami et al. 2021 |
| Perkinsozoa (Alveolata) | Reñé et al. 2017 |
| Syndiniales (Alveolata) | Jephcott et al. 2016 |
| Oomycota (Stramenopiles) | Buaya and Thines 2020 |
| Pirsoniales (Stramenopiles) | Kühn et al. 1996 |
| Cercozoa (Rhizaria) | Schepf and Kühn 2000, Kühn et al. 2000 |
| Labyrinthulomycetes  (Thraustochytrids and Labyrinthulids) | Raghukumar 2000, Hassett 2000 |

**Table S2:** Contribution of site, sampling date and size fraction to variation in **(a)** eukaryotic, **(b)** fungi, and **(c)** chytrid communities. PERMANOVA was applied for the

correlation between community compositions (Bray–Curtis dissimilarity distance) and each variable.

| **a) Eukaroytic Community** | | | | | | |
| --- | --- | --- | --- | --- | --- | --- |
|  | **Df** | **Sum of Sqs** | **R2** | **F** | **Pr(>F)** |  |
| **Site** | 1 | 2.1806 | **0.16874** | 9.8678 | ***0.001*** | ******* |
| **SamplingDate** | 2 | 1.7031 | **0.13179** | 3.8535 | ***0.001*** | ******* |
| **Size fraction** | 1 | 1.0462 | **0.08096** | 4.7344 | ***0.001*** | ******* |
| **Site:SamplingDate** | 2 | 1.3997 | **0.10831** | 3.1671 | ***0.001*** | ******* |
| **Site:Size fraction** | 1 | 0.4144 | **0.03206** | 1.8751 | ***0.027*** | ***** |
| SamplingDate:Size fraction | 2 | 0.4444 | 0.03439 | 1.0056 | 0.461 |  |
| Site:SamplingDate:Size fraction | 2 | 0.4309 | 0.03334 | 0.9749 | 0.508 |  |
| Residual | 24 | 5.3035 | 0.4104 |  |  |  |
| Total | 35 | 12.9228 | 1 |  |  |  |

| **b) Fungal Community** | | | | | | |
| --- | --- | --- | --- | --- | --- | --- |
|  | **Df** | **Sum of Sqs** | **R2** | **F** | **Pr(>F)** |  |
| **Site** | 1 | 1.8263 | 0.13680 | 11.3849 | ***0.001*** | ******* |
| **SamplingDate** | 2 | 3.4138 | 0.25572 | 10.6407 | ***0.001*** | ******* |
| **Size fraction** | 1 | 0.3868 | 0.02898 | 2.4114 | ***0.017*** | ***** |
| **Site:SamplingDate** | 2 | 3.1553 | 0.23636 | 9.8352 | ***0.001*** | ******* |
| **Site:Size fraction** | 1 | 0.1873 | 0.01403 | 1.1679 | 0.310 |  |
| SamplingDate:Size fraction | 2 | 0.2828 | 0.02118 | 0.8815 | 0.594 |  |
| Site:SamplingDate:Size fraction | 2 | 0.2477 | 0.01855 | 0.7721 | 0.736 |  |
| Residual | 24 | 3.8499 | 0.28838 |  |  |  |
| Total | 35 | 13.3499 | 1 |  |  |  |

| **c) Chytrid Community** |  |  |  |  |  |  |
| --- | --- | --- | --- | --- | --- | --- |
|  | **Df** | **Sum of Sqs** | **R2** | **F** | **Pr(>F)** |  |
| **Site** | 1 | 1.6844 | **0.12835** | 13.2732 | ***0.001*** | *** |
| **SamplingDate** | 2 | 3.887 | **0.2962** | 15.3151 | ***0.001*** | *** |
| Size fraction | 1 | 0.0781 | 0.00595 | 0.6152 | 0.734 |  |
| **Site:SamplingDate** | 2 | 4.179 | **0.31845** | 16.4656 | ***0.001*** | *** |
| Site:Size fraction | 1 | 0.0624 | 0.00475 | 0.4916 | 0.816 |  |
| SamplingDate:Size fraction | 2 | 0.0955 | 0.00728 | 0.3762 | 0.982 |  |
| Site:SamplingDate:Size fraction | 2 | 0.0911 | 0.00694 | 0.3589 | 0.984 |  |
| Residual | 24 | 3.0456 | 0.23208 |  |  |  |
| Total | 35 | 13.123 | 1 |  |  |  |

**Table S3:** Correlations of different combinations of environmental variables with a) eukaryotic, b) fungal and c) chytrid community

| a) Eukaryotic Community |  | b) Fungal Community |  |
| --- | --- | --- | --- |
| Environmental variables | Correlation | Environmental variables | Correlation |
| *Salinity* | *0.6023* | NH_4_^+^.N | 0.6626 |
| Salinity + Tot-P | 0.5779 | *Chl-a + NH_4_^+^.N* | *0.7698* |
| Salinity + Si + Tot-P | 0.5653 | Chl-a + NH_4_^+^.N + Tot-P | 0.7436 |
| Salinity + pH + Si+ Tot-P | 0.5590 | pH + Chl-a + NH_4_^+^.N + Tot-P | 0.7184 |
| Temp + Salinity + pH + Si + Tot-P | 0.5452 | Temp + pH + Chl-a + NH_4_^+^.N + Tot-P | 0.7181 |
| Temp + Salinity + pH + Chl-a + Si + Tot-P | 0.5052 | Temp + pH + Chl-a + NH_4_^+^.N + Tot-P + Tot-N | 0.6891 |
| Temp + Salinity + pH + Chl-a + NH_4_^+^.N + Si + Tot-P | 0.4767 | Temp + pH + Chl-a + NH_4_^+^.N + Si Tot-P + Tot-N | 0.6544 |
| Temp + Salinity + pH + Chl-a + NH_4_^+^.N + Si + Tot-P +Tot-N | 0.4411 | Temp + Salinity + pH + Chl-a + NH_4_^+^.N + Si Tot-P + Tot-N | 0.6174 |
| Temp + Salinity + pH + O_2_ + Chl-a + NH_4_^+^.N + Si+ Tot-P +Tot-N | 0.4016 | Temp + Salinity + pH + O_2_ + Chl-a + NH_4_^+^.N + Si Tot-P Tot-N | 0.5840 |

| c) Chytrid Community |  |
| --- | --- |
| Environmental variables | Correlation |
| NH4+.N | 0.5868 |
| *Chl-a + NH4+.N* | *0.7279* |
| Chl-a + NH4+.N + Tot-P | 0.6944 |
| pH Chl-a + NH4+.N + Tot-P | 0.6670 |
| Temp + pH + Chl-a + NH4+.N + Tot-P | 0.6529 |
| Temp + pH + Chl-a + NH4+.N + Tot-P + Tot-N | 0.6420 |
| Temp + pH + Chl-a + NH4+.N + Si + Tot-P + Tot-N | 0.6103 |
| Temp + Salinity + O2 + Chl-a + NH4+.N + Si Tot-P + Tot-N | 0.5800 |
| Temp + Salinity + pH + O2 + Chl-a + NH4+.N + Si + Tot-P + Tot-N | 0.5526 |
